# Efficient estimation of grouped survival models

**DOI:** 10.1186/s12859-019-2899-x

**Published:** 2019-05-28

**Authors:** Zhiguo Li, Jiaxing Lin, Alexander B. Sibley, Tracy Truong, Katherina C. Chua, Yu Jiang, Janice McCarthy, Deanna L. Kroetz, Andrew Allen, Kouros Owzar

**Affiliations:** 1 Department of Biostatistics and Bioinformatics, Duke University, Durham, USA; 2 Duke Cancer Institute, Duke University, Durham, USA; 3 Department of Bioengineering and Therapeutic Sciences, University of California San Francisco, San Francisco, USA

**Keywords:** Grouped data, Discrete censoring, Score statistic, Efficient score, Genome-wide analysis, Multiple testing, Heritability, Pharmacogenomics

## Abstract

**Background:**

Time- and dose-to-event phenotypes used in basic science and translational studies are commonly measured imprecisely or incompletely due to limitations of the experimental design or data collection schema. For example, drug-induced toxicities are not reported by the actual time or dose triggering the event, but rather are inferred from the cycle or dose to which the event is attributed. This exemplifies a prevalent type of imprecise measurement called grouped failure time, where times or doses are restricted to discrete increments. Failure to appropriately account for the grouped nature of the data, when present, may lead to biased analyses.

**Results:**

We present groupedSurv, an R package which implements a statistically rigorous and computationally efficient approach for conducting genome-wide analyses based on grouped failure time phenotypes. Our approach accommodates adjustments for baseline covariates, and analysis at the variant or gene level. We illustrate the statistical properties of the approach and computational performance of the package by simulation. We present the results of a reanalysis of a published genome-wide study to identify common germline variants associated with the risk of taxane-induced peripheral neuropathy in breast cancer patients.

**Conclusions:**

groupedSurv enables fast and rigorous genome-wide analysis on the basis of grouped failure time phenotypes at the variant, gene or pathway level. The package is freely available under a public license through the Comprehensive R Archive Network.

**Electronic supplementary material:**

The online version of this article (10.1186/s12859-019-2899-x) contains supplementary material, which is available to authorized users.

## Background

In basic science and translational studies, time-to-event outcomes are commonly measured imprecisely or incompletely due to limitations in the design or the data collection schema. For example, in mouse studies, due to practical and cost considerations, tumor progression events are not monitored in real time. Each event is recorded as occurring between two contiguous assessments. In clinical studies, drug-induced adverse events are commonly not reported by the date of occurrence but rather by drug cycle. These are examples of grouped failure time data, also referred to as grouped survival data, grouped censored data, or simply grouped data.

It should be noted that grouped failure time phenotypes are not restricted to studies with time-to-event outcomes. Cell-line cytotoxicty studies (e.g., growth-inhibitory effects of tamoxifen on human breast cancer cell lines [1]) are commonly designed on the basis of a pre-specified set of doses. Events, such as the half-maximal inhibitory concentration (IC50), are not directly observable due to the discrete nature of the dose design. The event of interest is observed to occur between two consecutive doses. In the subsequent report, we do not distinguish between time-to-event data and dose-to-event data, and refer to them as failure time data or survival time data.

One approach for dealing with grouped data is to use methods designed to adjust for tied continuous survival times in the presence of right censoring. Commonly used methods are those proposed by Breslow and Peto [2] or Efron [3]. As we will demonstrate in this paper, these methods may be biased when applied to grouped survival data. Another approach is to use an exact likelihood [4]. However, to do so is computationally intensive and thus not feasible for genome-wide inference.

In this paper, we present groupedSurv, an open source R [5] package implementing a statistically rigorous and computationally efficient approach proposed in Prentice and Gloeckler [6] for genome-wide analysis with group censored phenotypes. The package conducts analyses at a variant, gene or pathway level. Through applying groupedSurv to simulated survival data, we establish that our approach controls type I error and yields unbiased effect size estimates. We also show, by simulation, that when methods designed for tied survival outcomes are applied to grouped survival data, the results may be biased. On the basis of results from computational cost benchmarking studies on both real and simulated survival data, we demonstrate that our package completes analyses of large genome-wide association studies (GWAS), e.g., 1,000,000 variants and *n*=1000 samples, within three minutes on a four-core computer. To illustrate the application of our package, we conduct an analysis of a previously published GWAS to identify common variants associated with taxane-induced peripheral neuropathy in breast cancer patients [7].

## Implementation

### Statistical considerations

#### Statistical model

Under the standard Cox proportional hazards model [8], the conditional hazard function at time *t*>0, given variables **x**_*i*_ and **z**_*i*_, is canonically presented as

1 $$ \lambda(t|\mathbf{x}_{i},\mathbf{z}_{i})=\lambda_{0}(t)\exp\left(\mathbf{x}_{i}^{T} \mathbf{\beta} + \mathbf{z}_{i}^{T} \theta\right),  $$

where *λ*_0_(*t*) is an unspecified baseline hazard function, **x**_*i*_ is a vector of variables of interest, and **z**_*i*_ is a vector of baseline covariates. Here, **β** is the parameter vector of interest while *θ* is a nuisance parameter vector. Under the grouped failure time model, the event of interest will fall into one of *r* pre-specified time intervals, or be right-censored at the beginning of one of these intervals, denoted [*t*_*j*−1_,*t*_*j*_), for *j*∈{1,2,…,*r*}, where *t*_0_=0 and *t*_*r*_=*∞*. The groupedSurv package employs the regression model for grouped survival data proposed by Prentice and Gloeckler [6], which discretizes the conditional hazard function (1) according to these intervals. Additional technical details for the model are provided in the “Statistical model” section of Additional file 1.

#### Efficient score statistic

For a single variable of interest, *x*_*i*_, groupedSurv tests the hypothesis *H*_0_:*β*=0 against *H*_1_:*β*≠0, under the grouped failure time model. The test is conducted using an efficient score statistic [9] on the basis of the observed data, {**y**_1_=(*k*_1_,*δ*_1_,*x*_1_,**z**_1_),…,**y**_*n*_=(*k*_*n*_,*δ*_*n*_,*x*_*n*_,**z**_*n*_)}. Here, for sample *i*∈{1,…,*n*}, *k*_*i*_∈{1,2,…,*r*} denotes the interval of the observed grouped outcome and *δ*_*i*_∈{0,1} denotes the event indicator. In this notation, *k*_*i*_=*j*,*δ*_*i*_=1 indicates that the event occured in interval [*t*_*j*−1_,*t*_*j*_) while *k*_*i*_=*j*,*δ*_*i*_=0 indicates that the sample was right-censored at time *t*_*j*−1_. Using this notation, the corresponding likelihood function is

2 $$ L_{i}(\mathbf{y}_{i},\beta,\eta) = \left(1 - \alpha_{k_i}^{\exp(x_{i} \beta + \mathbf{z}_{i}^{T} \theta)} \right)^{\delta_i} \prod_{j=1}^{k_{i}-1} \alpha_{j}^{\exp\left(x_{i} \beta + \mathbf{z}_{i}^{T} \theta\right)},  $$

where $\alpha _{j} = \exp \left (-\int _{t_{j-1}}^{t_{j}} \lambda (u)\,du\right)$ and *η*=(*α*,*θ*).

Our inferential approach differs from that described in Prentice and Gloeckler in that we use a partitioned score statistic. More specifically, rather than testing the null hypothesis that all of the effect parameters are zero (*H*_0_:*β*=0 & *θ*=**0**), we are testing only the effect of *x*_*i*_ (*H*_0_:*β*=0), while treating the parameters of the baseline covariates, *θ* (as well as the *α*_*j*_) as nuisance parameters. Using the efficient score function for *β* in the above model, we define our efficient score statistic as

3 $$ \mathcal W = \frac{\left(\sum\limits_{i=1}^{n} S_{{\beta}}({0,\hat{{\eta}}})\right)^{2}} {n\left(\bar{\mathcal I}_{{\beta\beta}}({0,\hat{{\eta}}})- \bar{\mathcal I}_{{\beta\eta}}({0,\hat{{\eta}}})\bar{\mathcal I}_{{\eta\eta}}({0,\hat{{\eta}}})^{-1} \bar{\mathcal I}_{{\beta\eta}}({0,\hat{{\eta}}})^{T}\right)},  $$

where $\hat {\eta }$ is the maximum likelihood estimate (MLE) of the nuisance parameter under the null hypothesis, $ S_{{\beta }}({0,\hat {{\eta }}})$ is the score function with respect to *β*, $\bar {\mathcal I}_{{\beta \eta }}({0,\hat {{\eta }}})$ is the second derivative of the score statistic with respect to *β* and *η* (similarly for $\bar {\mathcal I}_{{\beta \beta }}({0,\hat {{\eta }}})$ and $\bar {\mathcal I}_{{\eta \eta }}({0,\hat {{\eta }}})$), and *n* is the sample size. The asymptotic null distribution for the statistic is chi-square, with degrees of freedom equal to the dimension of *β*. The derivation of this equation and other technical details, including our approach for approximating the standard error of the score statistic, are provided in Additional file 1.

#### Gene- and pathway-level statistics

Within the context of GWAS, what is often of interest is to conduct the analysis at the level of a gene or pathway rather than an individual variant. To allow for flexibility in conducting these types of set-based analyses, groupedSurv(), the primary function in the groupedSurv package, optionally returns the contribution of each sample to the score statistic for each variant tested. This enables users to employ the set-based statistic of their choice. An additional function, geneStat(), accepts a user-specified function as an argument and computes the gene- or pathway-level statistics directly. If no function is specified, geneStat() implements a sequence kernel association test (SKAT) [10, 11] statistic by default. Technical details are provided in the “Gene- and pathway-level statistics” section of Additional file 1.

#### Multiple testing

In addition to the unadjusted marginal asymptotic *P*-values for the variables tested, groupedSurv returns family-wise error rate (FWER) adjusted *P*-values and local false-discovery rates (FDR). The FWER-adjusted *P*-values are calculated based on the Bonferroni correction [12], while the local FDRs are based on Storey’s *Q*-values [13, 14].

### Software package

#### Design

The computational algorithms of groupedSurv are coded in C++ [15], and the Rcpp [16] package is used to interface with the R environment. The Rcpp package provides a series of R wrapper classes to import and load C++ code, and allows passing of R objects between R and C++. The pthreads model is used for implementing multi-threading. In addition to the help files for its functions, a vignette is provided as a user tutorial.

#### Input data format

The package is designed to accept multiple input data formats, including standard R data frames and matrix objects, and gwaa.data objects from the GenABEL package [17]. Data can also be imported from binary PLINK [18] files using the BEDMatrix package [19]. Genotype dosage data, obtained from imputation software (e.g., MACH [20, 21] or IMPUTE [22]) in the form of VCF [23] files, can be imported using the VariantAnnotation package [24]. The package vignette provides examples for importing each of the data formats. Note that, in the case of missing genotypes or genotype dosage data the package implements a complete-case analysis of each variant.

#### Usage

The primary function of the package, groupedSurv(), is capable of executing multiple analyses in parallel. It returns a data frame containing the efficient score statistics, along with the (unadjusted) asymptotic *P*-values, FWER-adjusted *P*-values, and the FDR for each of the variables of interest.

The thetaEst() function provides MLEs for the nuisance parameters, i.e., the baseline survival rates for each interval, $\hat {{\alpha }}_{j}$, and the parameters for any covariates, $\hat {{\theta }}$. The estimates are computed under the null hypothesis, i.e., that the variable of interest has no effect on the time to event. This allows these estimates to be reused to calculate the efficient score statistic for any number of variables being tested.

### Evaluation

#### Survival data simulation

We evaluate the accuracy and computational costs of the implemented methods using simulations. Grouped survival data are simulated by first generating continuous survival times, and then translating these into grouped survival times. First, the variables of interest, *X*, and two covariates, *Z*=(*Z*_1_,*Z*_2_), are simulated. Conditional on the realized values *X*=*x* and *Z*=(*z*_1_,*z*_2_), the continuous survival time, *T*, is drawn from an exponential distribution with hazard rate exp(*βx*+*θ*_1_*z*_1_+*θ*_2_*z*_2_). The censoring time, *C*, is drawn from a uniform distribution over (0,*c*_max_).

To transform continuous times into grouped times, we first specify a final observation time point *τ*. The interval [0,*∞*) is then divided into *r* contiguous intervals, composed of *r*−1 finite intervals of equal size spanning [0,*τ*), and a final *r*^th^ interval, [*τ*,*∞*). The resulting right-end points of the finite intervals represent the study observation time points. We define *T*^∗^ and *C*^∗^ as the right-end points of the intervals containing *T* and *C*, respectively. Grouped survival time is then defined as $\tilde {T}=\min (T^{\ast },C^{\ast })$, and the event indicator is given by $\mathbbm {1}\{T^{\ast }<C^{\ast }~\&~\tilde {T} \le \tau \}$. Technical details for the simulation approach are provided in the “Simulation” section of Additional file 1.

#### Statistical operating characteristics

We assess the statistical operating characteristics of our approach on the basis of empirical type I error control, statistical power, and bias of the effect size estimates. We also compare these characteristics to those of the Cox proportional hazards model [8], using either the Efron or exact likelihood methods to adjust for tied survival times in the presence of right censoring. We use the implementations of these approaches provided by the survival [25] R extension package.

#### Benchmarking

We conduct additional simulation studies to assess the computational performance of groupedSurv. To this end, we consider a range of sample sizes and variable counts. We also assess the performance gains from increasing the number of CPU cores used for parallel processing. Ten thousand replicate simulations are conducted for each scenario. The benchmarking analyses are performed on a AMD Opteron^TM^ 6180 SE Server CPU running the Debian Stretch (9.3) AMD64 GNU/Linux. A detailed description of the parameters of each simulation are provided in the “Simulation” section of Additional file 1.

#### Reproducible pipeline

The knitr [26] R extension package is used to reproducibly conduct the data simulation, summarize the operating characteristics, and estimate the processing benchmarks. The scripts to reproduce the simulation and operating characteristics are provided as Additional file 2.

### Data analysis

#### CALGB 40101 clinical and GWAS data

CALGB 40101 is a randomized phase III study comparing the efficacy of two standard adjuvant therapy regimens in women with breast cancer. It employs a two-by-two factorial design, randomizing patients to paclitaxel versus doxorubicin and cyclophosphamide, and four versus six cycles of therapy. Blood samples from patients who consent to participate in a pharmacogenomic companion study (CALGB 60202) and provide usable DNA are genotyped on the Illumina 610 quad platform. A genome-wide analysis to identify single nucleotide polymorphisms (SNPs) associated with paclitaxel-induced peripheral neuropathy is reported by Baldwin et al. [7]. The authors employ a cumulative dose to first grade 2 or higher paclitaxel-induced peripheral neuropathy event as the phenotype and conduct the analysis using the Cox score statistic for right-censored outcomes. The Efron approximation is used to deal with any ties in the cumulative dose outcome.

The clinical and genomic data are available for download from the database of Genotypes and Phenotypes (dbGaP) through accession phs000807.v1.p1. Additional details about the clinical study are provided in Shulman et al. [27]. Additional details about the GWAS data and neuropathy phenotyping are provided in Baldwin et al. [7].

#### Analysis of the CALGB 40101 data using groupedSurv

The study population for our analysis consists of 859 genetically estimated European patients identified in the CALGB 40101 GWAS publication. Four patients with no outcome data are uninformatively censored at time 0, effectively excluding them from the analyses. An addition 11 patients were missing baseline covariates or paclitaxel dosing information, and were also excluded.

After limiting the population to genetically estimated European patients with complete data, SNPs with call rates < 95*%*, Hardy-Weinberg *P*-values < 10^−8^, or relative minor allele frequency (MAF) < 0.05 are removed. Analyses are limited to autosomal SNPs only. The filtering is conducted using the GenABEL [17] package. The final genome-wide analysis is conducted across 500,897 SNPs.

#### Study model

SNPs are tested, under the additive genetic model, for association with cumulative dose to paclitaxel-induced peripheral neuropathy. Cumulative dose is measured as the number of cycles of paclitaxel received (1,2,3,4,5 or 6) prior to neuropathy event or treatment termination. For the analyses, body surface area (BSA) at clinical baseline and age at registration (log base 10 transformed) are used as covariates. GWAS is conducted both with groupedSurv and the coxph() function from the survival [25] package (using the Efron method for ties), and the top hits, ranked by unadjusted *P*-values from groupedSurv, are compared. The scripts to reproduce the analyses are provided as Additional file 3.

Manhattan and quantile-quantile (QQ) plots are used to visualize the empirical distribution of the resulting unadjusted asymptotic *P*-values. To illustrate the effect size, we use the non-parametric MLEs of the survival function [28] for interval-censored outcomes provided by the icfit() function from the interval [29] package. For regional visualizations, LocusZoom [30] plots are generated for the selected SNPs or genes.

## Results

### Statistical operating characteristics

**Fig. 1:**
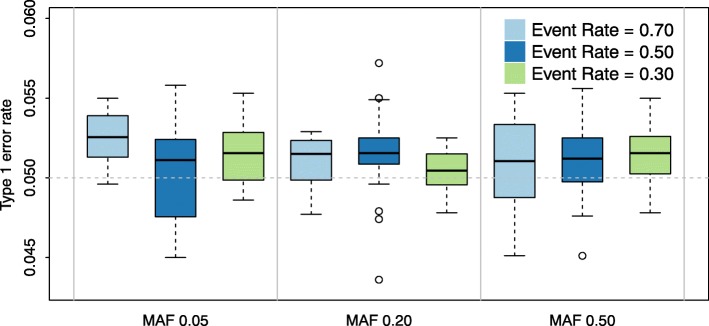
Box Plot for Type 1 Error Box plot of type 1 error of groupedSurv for different event rates and minor allele frequencies

We establish the statistical operating characteristics for our approach by empirical assessment of the type I error and the bias of the effect size for the simulated variant and the nuisance parameters. The type I error simulation results are shown in Fig. 1 for MAFs of 0.05,0.2 and 0.5, and event rates of 0.3, 0.5 and 0.7. Each example is based on a sample size of *n*=1000 and *B*=10,000 simulation replicates. These results provide confirmation that our approach provides type I error control.

**Fig. 2:**
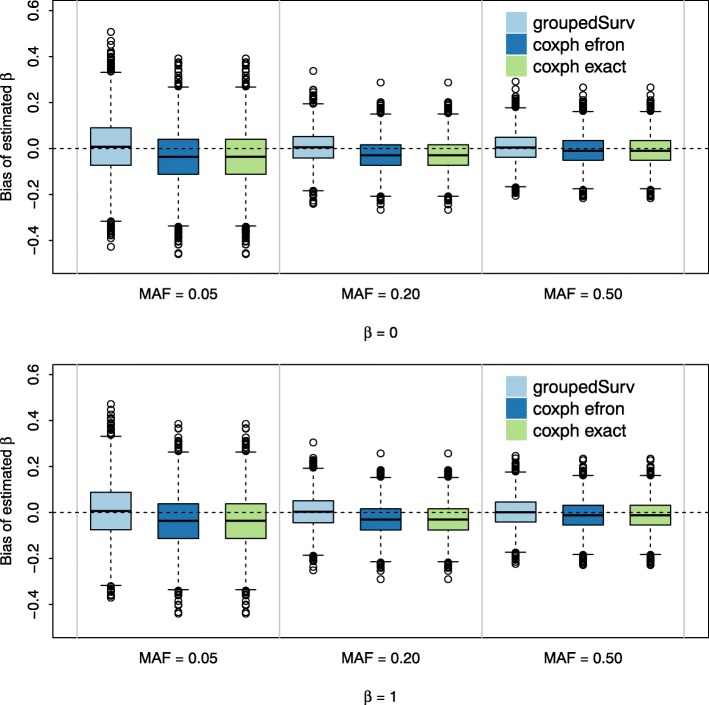
Effect Size Estimation Bias for groupedSurv and Coxph Box plot comparing bias of effect size estimation of groupedSurv and Coxph

The simulation results for the empirical bias assessments are shown in Fig. 2 for MAFs of 0.05,0.2 and 0.5. An event rate of 0.6 and a sample size of *n*=1000 is used for each example, under both the null (effect size *β*=0) and alternative (effect size *β*=1) hypotheses. Along with the results from our approach, we provide the corresponding results using the Efron and exact methods of adjusting for ties in a standard right-censored analysis. Each example consists of *B*=10,000 replicates. Our approach produces evidently unbiased estimates, regardless of MAF. The other two approaches seemingly underestimate the effect size for MAFs of 0.05 and 0.2, while producing unbiased estimates only in the most statistically powerful case when the MAF is 0.5 [31].

**Fig. 3:**
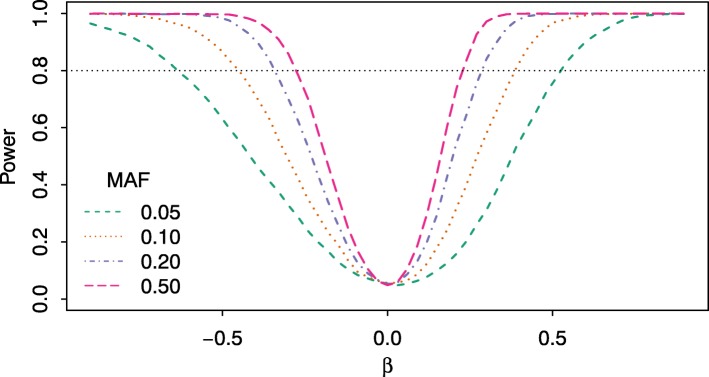
Power for groupedSurv Power estimates for groupedSurv for different minor allele frequencies

Having established control of type I error, the simulation results for the power estimations for the grouped failure time method are shown in Fig. 3 for MAFs of 0.05, 0.1, 0.2 and 0.5. An event rate of 0.6 and a sample size of *n*=1000 is used for each example, with the effect size varying over the range of *β*∈(−0.9,0.9). Power is estimated at the two-sided *α*=0.05 level using *B*=10,000 replicates.

These assessments of the statistical operating characteristics, bias, type I error, and power are also repeated for a smaller sample size (*n*=500). The results are included in Additional file 1.

### Computational performance

**Fig. 4:**
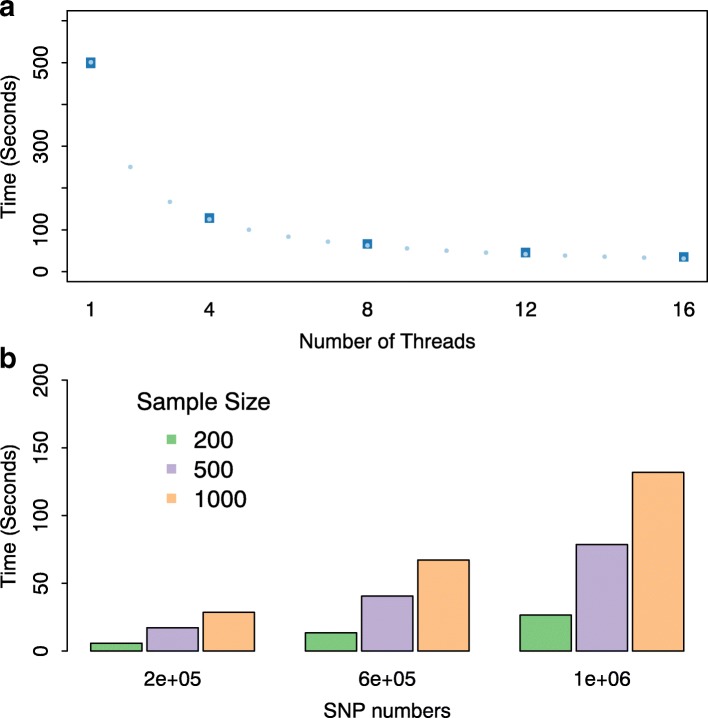
Timing benchmark plots for groupedSurv for **a** different numbers of CPU cores, and **b** different sample sizes and numbers of SNPs

To assess the computational performance of groupedSurv in terms of parallel efficiency, we consider a sample size of *n*=1,000, with 100,000 simulated SNPs to be tested individually as the variables of interest. The timing results based on 1, 4, 8, 12 and 16 CPU cores are shown in panel (a) of Fig. 4. The squares represent the observed CPU time using multiple cores while the circles represent the ideal performance, calculated as CPU time using one core divided by the number of CPU cores in each scenario.

To evaluate the performance of groupedSurv in terms of sample size and number of variables tested, we consider sample sizes of *n*=100, 500 and 1000, and SNP counts of 200,000, 500,000 and 1,000,000. The models also include two baseline covariates. The computational costs for the different sample sizes and numbers of SNPs are shown in panel (b) of Fig. 4, based on four CPU cores.

### CALGB 40101 GWAS results

**Fig. 5:**
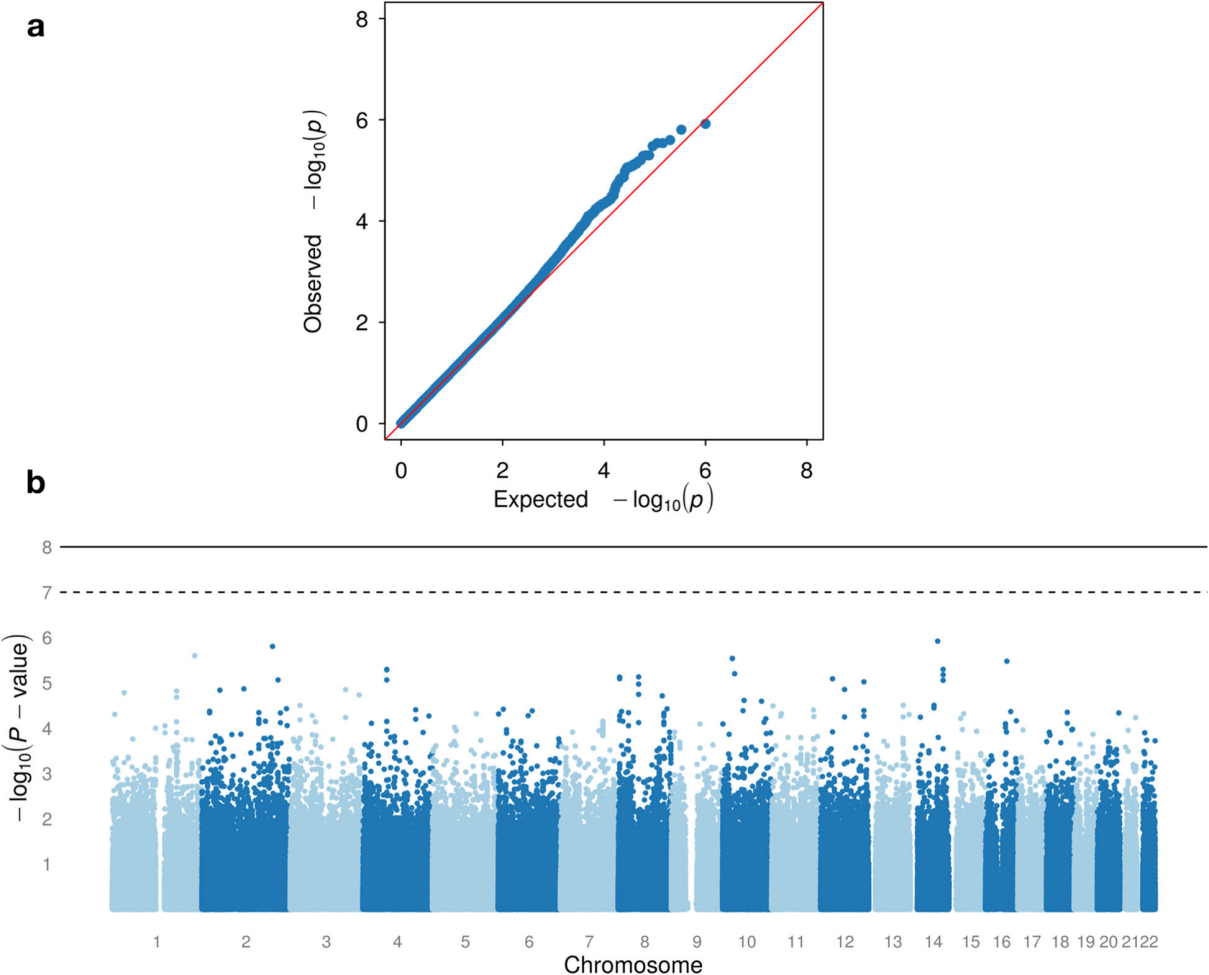
QQ and Manhattan Plot for GWAS(**a**) Quantile-quantile plot of expected − log10(*P*-values) and observed − log10(*P*-values) for all SNOs analyzed, (**b**)Manhattan plot of − log10(*P*-values) for all SNPs analyzed

The analysis is based on outcome data from 844 CALGB 40101 patients with 500,897 SNPs passing quality controls. An annotated list of the top 300 SNPs, ranked by unadjusted *P*-value, is provided as Additional file 4. The QQ and Manhattan plots for all SNPs are shown in Fig. 5. The QQ plot does not exhibit evidence of inflation.

**Fig. 6:**
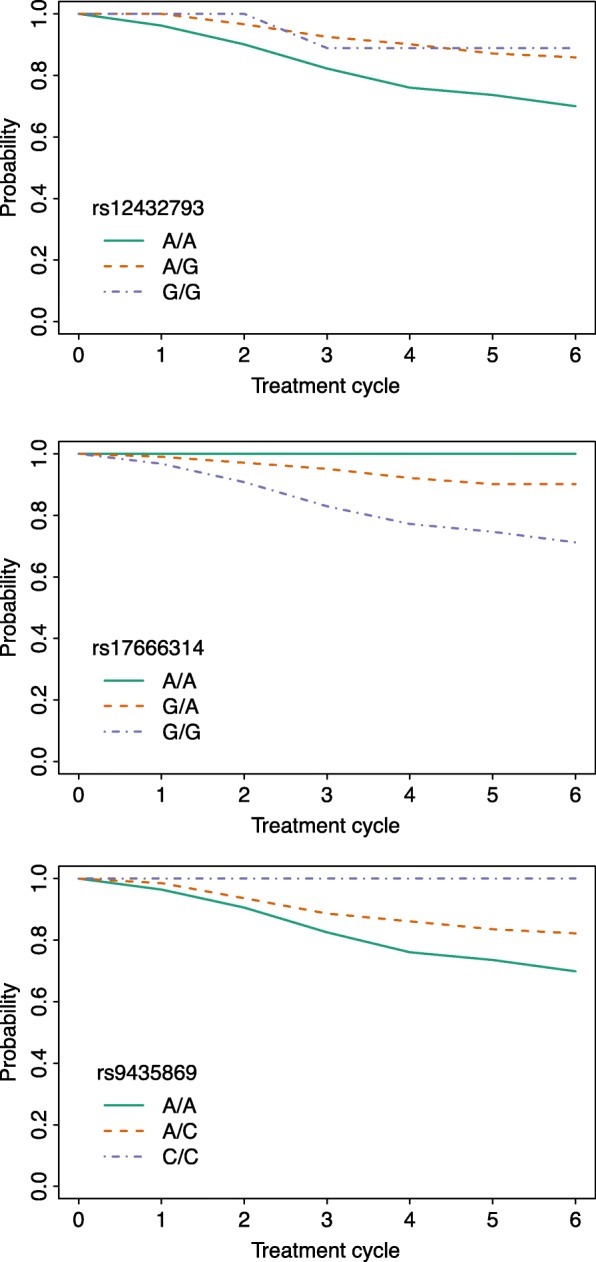
Non-parametric Maximum Likelihood Survival Survival plots for the top three SNPs

None of the 500,897 variants meet the genome-wide threshold of significance of 1 × 10^−8^. We prioritize the variants according to their corresponding unadjusted *P*-values for further examination with respect to potential biological relevance. The top 50 hits are shown in Table 1. Figure 6 shows the non-parametric MLEs of neuropathy-free survival by genotype for the top three hits. Survival and regional visualization plots are provided for six additional SNPs in Additional file 1.

**Table 1:** Top hits from CALGB 40101

rsID	Chr:Position	Annotation^*^	Location	MAF	*P*-value	$\hat {\beta }$
rs12432793	14:75631922	*FLVCR2*	promoter	0.12	1.2×10^−6^	-0.81
rs17666314	2:193496633	*PCGEM1/LINC01821*	intergenic	0.063	1.6×10^−6^	-1.1
rs9435869	1:229252924	*RHOU/RAB4A*	intergenic	0.15	2.5×10^−6^	-0.67
rs11015445	10:27001232	*ANKRD26*	intron	0.052	2.9×10^−6^	-1.1
rs4747583	10:27055768	*ANKRD26*	intron	0.052	2.9×10^−6^	-1.1
rs1035538	16:60045258	*LINC02141*	intron	0.064	3.3×10^−6^	-0.99
rs11627718	14:90630200	*TTC7B*	intron	0.12	5×10^−6^	-0.74
rs9684260	4:65303925	*LINC02232/EPHA5*	intergenic	0.36	5.1×10^−6^	0.48
rs7349683	4:65332086	*EPHA5*	coding	0.36	5.2×10^−6^	0.48
rs6481837	10:33156436	*ITGB1/NRP1*	intergenic	0.39	6.3×10^−6^	-0.45
rs1286439	14:90632867	*TTC7B*	intron	0.12	6.7×10^−6^	-0.74
rs4737264	8:55198762	*XKR4*	intron	0.21	7.4×10^−6^	0.53
rs9785155	8:4312184	*CSMD1*	intron	0.24	7.5×10^−6^	-0.54
rs10503252	8:4312237	*CSMD1*	intron	0.24	8.1×10^−6^	-0.54
rs10771973	12:32640040	*FGD4*	intron	0.32	8.1×10^−6^	0.46
rs1551124	4:65297857	*LINC02232/EPHA5*	intergenic	0.36	8.6×10^−6^	0.47
rs1517347	2:208649191	*PTH2R*	intron	0.27	8.6×10^−6^	-0.5
rs1286422	14:90647193	*LOC101928909*	intron	0.12	8.8×10^−6^	-0.72
rs10774538	12:119965993	*CIT/BICDL1*	intergenic	0.14	9.5×10^−6^	-0.64
rs1842501	8:55190458	*XKR4*	intron	0.21	1.1×10^−5^	0.53
rs6754133	2:114779214	*DPP10*	intron	0.056	1.4×10^−5^	-1
rs17781082	12:67082547	*LOC102724421*	intron	0.42	1.4×10^−5^	0.46
rs11924857	3:150955018	*CLRN1-AS1*	intron	0.21	1.4×10^−5^	-0.52
rs11680024	2:49308717	*FSHR/NRXN1*	intergenic	0.12	1.5×10^−5^	-0.71
rs11582158	1:179507372	*AXDND1*	intron	0.23	1.5×10^−5^	-0.51
rs12138026	1:33487056	*ZSCAN20*	intron	0.1	1.7×10^−5^	-0.74
rs6999054	8:55201958	*XKR4*	intron	0.21	1.8×10^−5^	0.51
rs1903216	3:187911715	*BCL6/LPP-AS2*	intergenic	0.48	1.9×10^−5^	0.44
rs13257404	8:120112872	*COL14A1*	intron	0.056	1.9×10^−5^	-1
rs12403933	1:179454485	*AXDND1*	intron	0.23	2.1×10^−5^	-0.5
rs3862864	10:58153109	*MIR3924/IPMK*	intergenic	0.088	2.4×10^−5^	-0.77
rs11192557	10:105692925	*SORCS3/LOC101927549*	intergenic	0.12	2.5×10^−5^	-0.66
rs17753508	14:65590734	*FUT8*	intron	0.19	3.1×10^−5^	-0.55
rs1729775	13:95105689	*ABCC4*	intron	0.41	3.1×10^−5^	-0.44
rs7616728	3:26664146	*LRRC3B*	intron	0.16	3.2×10^−5^	-0.57
rs7936678	11:3610877	*LOC101927708/ART5*	intergenic	0.21	3.2×10^−5^	-0.52
rs10483776	14:65448149	*FUT8*	intron	0.19	3.6×10^−5^	-0.55
rs4530357	2:231675992	*TEX44/PTMA*	intergenic	0.44	3.7×10^−5^	-0.41
rs2233335	8:133248822	*NDRG1*	intron	0.38	3.7×10^−5^	-0.44
rs12191315	NA:NA	*NA/NA*		0.19	3.8×10^−5^	-0.52
rs4599356	4:144202300	*GYPA/HHIP-AS1*	intergenic	0.39	3.9×10^−5^	-0.43
rs10891842	11:115368305	*CADM1*	intron	0.42	4×10^−5^	0.4
rs7295447	12:119925810	*CIT/BICDL1*	intergenic	0.17	4.1×10^−5^	-0.55
rs11004791	10:55267443	*PCDH15*	intron	0.47	4.1×10^−5^	-0.39
rs2169100	NA:NA	*NA/NA*		0.27	4.1×10^−5^	-0.45
rs6714773	2:21266211	*TDRD15/LINC01822*	intergenic	0.28	4.2×10^−5^	-0.49
rs1993596	8:9365634	*LOC157273*	intron	0.45	4.3×10^−5^	0.42
rs11075766	16:70529368	*SNORD111B*	promoter	0.078	4.3×10^−5^	-0.77
rs929525	18:61429532	*CDH20*	intron	0.25	4.5×10^−5^	-0.49
rs2141236	2:155938902	*NA/LINC01876*	intergenic	0.34	4.5×10^−5^	0.39

Top 50 hits, ranked by unadjusted *P*-value from analysis of 500,897 genome-wide SNPs. Annotation information was generated by using the VariantAnnotation [24], TxDb.Hsapiens.UCSC.hg38.knownGene [44] (based on [45]) and SNPlocs.Hsapiens.dbSNP150.GRCh38 [46] R extension packages. For intergenic SNPs, left/right flanking genes are reported. Chr: chromosome; MAF: minor allele frequency

## Discussion

On the basis of results from the application of the groupedSurv package to simulated survival data, we demonstrate the theoretical properties of our approach. More specifically, we confirm that the testing rule provides proper type I error control, and that the model estimates are asympotically unbiased.

The groupedSurv package enables fast genome-wide inference based on grouped censored phenotypes. As Fig. 4 illustrates, on a single core, the analysis of 1,000,000 variants and *n*=1000 patients requires less than 8.5 minutes. The completion times are reduced to about 2.1, 1.0, 0.7 or 0.5 minutes when increasing the core count to 4, 8, 12 or 16, respectively.

As we have outlined, an alternative approach to using a likelihood model for grouped data is to repurpose methods for adjustments for ties in continuous right censored data. As we and others [31] have shown, these methods may be biased if applied to grouped censored data. The effect of sample size on this bias is illustrated in Figure S1 in Additional file 1.

Although the analysis using this approach did not identify any variants that reached the genome-wide threshold for significance, the annotated results (Table 1) revealed additional candidate genes undiscovered in the initial GWAS. Among the genes listed in Table 1, many show functions in biological pathways critical to neurite regeneration after chemotherapy damage and show modest to high expression in human dorsal root ganglion, the target peripheral nerve damaged by chemotherapy agents [32]. In particular, the genomic region containing rs9435869 is an intriguing hit since both neighboring genes have potential relevance to chemotherapy-induced peripheral neuropathy (CIPN). *RHOU* is a Rho GTPase that is known to regulate cytoskeletal organization and induce filopodium formation, a critical step in neuronal development [33–35]. Furthermore, *RAB4A*, encoding a member of the RAB GTPase family, is a regulator of vesicular recycling of cell surface receptors through interaction with its effector *NDRG1* [36]. Mutations in *NDRG1* (N-myc downstream-regulated gene 1) have been shown to be causative in the rare congenital subtype of Charcot-Marie Tooth Disease Type 4D, an inherited peripheral sensorimotor nerve disorder [37–39].

Another SNP included in the top 50 hits, rs6481837, annotated to *NRP1* (neuropilin 1) which functions to control growth cone projection in developing neurons [40], supports the hypothesis that axon guidance is important in the development of CIPN. This gene pathway was previously implicated with the role of *EPHA5* from the initial GWAS [27]. Genes involved in synaptic formation associated to neurodevelopmental disorders of the central nervous system, such as *NRXN1* (neurexin 1) and *CADM1* (cell adhesion molecule 1), may also have biological relevance in the context of the peripheral nervous system during the manifestation of this drug-induced neurotoxicity [41, 42]. Although these genes/variants are promising for understanding the molecular mechanisms of CIPN, further in vitro investigations are needed.

Here we note several limitations in our approach. First, while the simulation results confirm that the MLEs are asymptotically unbiased, the effect size estimates are model-based and thus may be biased if the model is misspecified. Second, because the phenotype for the CALGB 40101 GWAS analysis is constructed via a manual review of clinical research forms [7], the cycle attribution may be erroneous due to incomplete or missing information. Our approach properly accounts for the group censoring mechanism, however, it may not be able to account for such phenotyping errors. Lastly, in the analysis of the CALGB 40101 data, we assume that the censoring mechanism is uninformative. Although the censoring induced by assignment to four versus six cycles can safely be assumed to be uninformative as it was decided by study randomization, other forms of censoring may be informative, such as early dropouts due to drug sensitivity. If informative censoring is present, the model parameters need to be interpreted within the context of cause-specific hazards.

We conclude this section by considering potential future extensions. In its current form, our approach does not allow for time-dependent covariates. For example, BSA at baseline is a covariate in our analysis of the CALGB 40101 GWAS. BSA, however, varies at each cycle. Prentice and Gloeckner [6] note that it is possible to extend the regression model to incorporate time-dependent covariates. Another extension, that is currently partially implemented, is the ability to analyze data from familial genetic studies, in which the outcomes may be correlated. The package incorporates a kinship frailty model [43], however, the current implementation is sensitive to departures from model assumptions. Therefore, at present we consider it as an experimental feature. A future extension of this implementation is to make it more robust.

## Conclusions

Grouped censored phenotypes are prevalent in basic, translational and clinical science research due to the design of, or limitations in, the data collection schema. Failure to properly account for a grouped failure time mechanism may lead to biased analysis results. We present groupedSurv, an open source R package for conducting genome-wide analyses based on grouped survival phenotypes in a statistically principled and rigorous, and computationally efficient manner. In the context of GWAS, the package enables analysis at the variant as well as gene or pathway level. The package is extensively documented and freely available under a public license to the research community.

## Availability and requirements

**Project name:** groupedSurv

**Project home page:** https://CRAN.R-project.org/package=groupedSurv

**Operating system(s):** Linux, Windows and OS X

**Programming language:** R

**Other requirements:**C++ 11 or higher, and supporting R packages required for installation

**License:** GPL (≥2)

**Any restrictions to use by non-academics:** None

## Additional files

Additional file 1 Additional descriptions of statistical model, simulation parameters, and statistical operating characteristics and data analysis results, including additional illustrative figures. (PDF 556 kb)

Additional file 2 knitr-generated slides showing R code used to reproducibly conduct the data simulations, summarize the operating characteristics, and estimate the processing benchmarks. (PDF 112 kb)

Additional file 3 knitr-generated slides showing R code used to reproducibly conduct the analysis of the CALGB 40101 data. (PDF 145 kb)

Additional file 4 Table containing the annotated top 300 SNPs, ranked by unadjusted P-value, from the CALGB 40101 GWAS results. (CSV 29 kb)

## Abbreviations

BSA: Body surface area
CIPN: Chemotherapy-induced peripheral neuropathy
FDR: False discovery rate
FWER: Family-wise error rate
GWAS: Genome-wide association study
IC50: Half-maximal inhibitory concentration
MAF: Minor allele frequency
MLE: Maximum likelihood estimate
QQ plot: Quantile-quantile plot
SKAT: Sequence kernel association test
SNP: Single nucleotide polymorphism
