# Supplementary material for: Efficient estimation of grouped survival models
Source: BMC Bioinformatics. 2019 May 28;20:269. doi: 10.1186/s12859-019-2899-x (PMC6540566; doi:10.1186/s12859-019-2899-x)
Supplement: Supplementary file 1 — Additional descriptions of statistical model, simulation parameters, and statistical operating characteristics and data analysis results, including additional illustrative figures. (PDF 556 kb) [file 12859_2019_2899_MOESM1_ESM.pdf]

Efficient Estimation of Grouped Survival Models

Zhiguo Li[1,2], Jiaxing Lin[1], Alexander B Sibley[2], Tracy Truong[1], Katherina C Chua[3], Yu Jiang[1], Janice McCarthy[1], Deanna L Kroetz[3], Andrew Allen[1], and Kouros Owzar[1,2]

[1]Department of Biostatistics and Bioinformatics, Duke University Medical Center, Durham, NC, USA
[2]Duke Cancer Institute, Duke University Medical Center, Durham, NC, USA
[3]Department of Bioengineering and Therapeutic Sciences, University of California San Francisco, San Francisco, CA, USA

Statistical model

Likelihood function

Suppose we have iid observations from n subjects. Let T_i denote the time to the event of interest for subject i. Also define $\mathbf{x}_i$, a vector of variables of interest, and $\mathbf{z}_i$, a vector of nuisance covariates. Assume the Cox proportional hazards model

$$\lambda(t|\mathbf{x}_i, \mathbf{z}_i) = \lambda_0(t) \exp(\mathbf{x}_i^T \beta + \mathbf{z}_i^T \theta),$$

where $\lambda(t|\mathbf{x}_i, \mathbf{z}_i)$ is the hazard function of T_i given $(\mathbf{x}_i, \mathbf{z}_i)$, and $\lambda_0(t)$ is the baseline hazard function. Given $(\mathbf{x}_i, \mathbf{z}_i)$, the survival function of T_i is

$$\mathsf{S}(t|\mathbf{x}_i, \mathbf{z}_i) = \mathsf{S}_0(t)^{\exp(\mathbf{x}_i^T \beta + \mathbf{z}_i^T \theta)} = \exp\Big(-\int_0^t \lambda_0(u)\, du\Big)^{\exp(\mathbf{x}_i^T \beta + \mathbf{z}_i^T \theta)}.$$

Now consider a pre-specified partition of $[0, \infty)$, $0 = t_0 < t_1 < t_2 < \cdots < t_r = \infty$, where r is a positive integer. For subject i, T_i is either observed to belong to the interval $[t_{k_i-1}, t_{k_i})$ or is censored at the beginning of $[t_{k_i-1}, t_{k_i})$, where $k_i \in \{1, 2, \ldots, r\}$. If T_i is observed to belong to such a finite interval, we define $\delta_i = 1$, and otherwise define $\delta_i = 0$. We denote the observed data vector as $\mathbf{y}_i = (k_i, \delta_i, \mathbf{x}_i, \mathbf{z}_i)$. An individual subject's contribution to the grouped survival likelihood function, based on equation (4) in [1], is

$$L_i(\mathbf{y}_i, \beta, \eta) = (1 - \alpha_{k_i}^{\exp(\mathbf{x}_i^T \beta + \mathbf{z}_i^T \theta)})^{\delta_i} \prod_{j=1}^{k_i-1} \alpha_j^{\exp(\mathbf{x}_i^T \beta + \mathbf{z}_i^T \theta)}$$

where $\alpha_j = \exp(-\int_{t_{j-1}}^{t_j} \lambda(u)\, du)$ and $\eta = (\gamma, \theta)$ is the vector of nuisance parameters.

The full likelihood would be the product of the L_i over all study subjects. The log-likelihood function then can be written as a sum over all subjects,

$$l(\beta, \eta) = \sum_{i=1}^{n} \left[\delta_i \log(1 - \exp(-\exp(\gamma_{k_i} + \mathbf{x}_i^T \beta + \mathbf{z}_i^T \theta))) - \sum_{j=1}^{k_i - 1} \exp(\gamma_j + \mathbf{x}_i^T \beta + \mathbf{z}_i^T \theta) \right]$$

where, following equation (5) in [1], we substitute $\gamma_j = \log(-\log(\alpha_j))$, to remove the range restrictions on the parameters.

Henceforth we consider the simple example where $\mathbf{x}_i = x_i$, a single explanatory variable of interest.

Score functions

$$S_\beta(\mathbf{y}_i, \beta, \eta) = \frac{\partial}{\partial \beta} l(\mathbf{y}_i, \beta, \eta) = x_i \left(c(\mathbf{y}_i, \beta, \eta) - \sum_{j=1}^{k_i - 1} \exp(\gamma_j + x_i \beta + \mathbf{z}_i^T \theta) \right),$$

$$S_{\theta_m}(\mathbf{y}_i, \beta, \eta) = \frac{\partial}{\partial \theta_m} l(\mathbf{y}_i, \beta, \eta) = z_{im} \left(c(\mathbf{y}_i, \beta, \eta) - \sum_{j=1}^{k_i - 1} \exp(\gamma_j + x_i \beta + \mathbf{z}_i^T \theta) \right), \text{ for covariate } m \text{ in } \mathbf{z}_i,$$

$$S_\gamma(\mathbf{y}_i, \beta, \eta) = \frac{\partial}{\partial \gamma} l(\mathbf{y}_i, \beta, \eta) = \begin{pmatrix} \frac{\partial}{\partial \gamma_1} l(\mathbf{y}_i, \beta, \eta) \\ \vdots \\ \frac{\partial}{\partial \gamma_r} l(\mathbf{y}_i, \beta, \eta) \end{pmatrix}, \text{ where}$$

$$\frac{\partial}{\partial \gamma_j} l(\mathbf{y}_i, \beta, \eta) = \begin{cases} -\exp(\gamma_j + x_i \beta + \mathbf{z}_i^T \theta) & j < k_i \\ c(\mathbf{y}_i, \beta, \eta) & j = k_i \\ 0 & j > k_i \end{cases}, \text{ for } j = 1, \ldots, r.$$

For readability we use the substitution $c(\mathbf{y}_i, \beta, \eta) = \delta_i \frac{\exp(-\exp(\gamma_{k_i} + x_i\beta + \mathbf{z}_i^T\theta)) \exp(\gamma_{k_i} + x_i\beta + \mathbf{z}_i^T\theta)}{1 - \exp(-\exp(\gamma_{k_i} + x_i\beta + \mathbf{z}_i^T\theta))}$.

Efficient score

Let $\mathcal{I}_{\beta\eta}(\mathbf{y}_i, \beta, \eta) = S_\beta(\mathbf{y}_i, \beta, \eta) S_\eta(\mathbf{y}_i, \beta, \eta)^T$, and $\mathbb{I}_{\beta\eta}(\beta, \eta) = \mathbb{E}\left[\mathcal{I}_{\beta\eta}(\mathbf{y}_i, \beta, \eta)\right]$, (similarly for $\mathcal{I}_{\beta\beta}(\mathbf{y}_i, \beta, \eta)$ and $\mathbb{I}_{\beta\beta}(\beta, \eta)$, and $\mathcal{I}_{\eta\eta}(\mathbf{y}_i, \beta, \eta)$ and $\mathbb{I}_{\eta\eta}(\beta, \eta)$). The contribution of an individual subject to the efficient score [2] for the parameter of interest β is defined as

$$\mathbb{U}(\mathbf{y}_i, \beta, \eta) = S_\beta(\mathbf{y}_i, \beta, \eta) - \mathbb{I}_{\beta\eta}(\beta, \eta) \mathbb{I}_{\eta\eta}(\beta, \eta)^{-1} S_\eta(\mathbf{y}_i, \beta, \eta).$$

We evaluate the efficient score at $\beta = 0$ and $\eta = \hat{\eta}$, where $\hat{\eta}$ the MLE of the nuisance parame-

ters estimated under the null hypothesis.

$$\begin{aligned}
\mathbb{U}(0,\hat{\eta}) &= \sum_{i=1}^{n} \mathbb{U}(\mathbf{y}_i, 0, \hat{\eta}) \\
&= \sum_{i=1}^{n} \left(S_\beta(\mathbf{y}_i, 0, \hat{\eta}) - \mathbb{I}_{\beta\eta}(0,\hat{\eta})\mathbb{I}_{\eta\eta}(0,\hat{\eta})^{-1} S_\eta(\mathbf{y}_i, 0, \hat{\eta}) \right) \\
&= \sum_{i=1}^{n} S_\beta(\mathbf{y}_i, 0, \hat{\eta}) - \mathbb{I}_{\beta\eta}(0,\hat{\eta})\mathbb{I}_{\eta\eta}(0,\hat{\eta})^{-1} \sum_{i=1}^{n} S_\eta(\mathbf{y}_i, 0, \hat{\eta}) \\
&= \sum_{i=1}^{n} S_\beta(\mathbf{y}_i, 0, \hat{\eta}),
\end{aligned}$$

as $\sum_{i=1}^{n} S_\eta(\mathbf{y}_i, 0, \hat{\eta}) = 0$ since $\hat{\eta}$ is the MLE under H_0.

The efficient score statistic for testing $H_0 : \beta = 0$ can be calculated [2] as

$$\begin{aligned}
\mathbb{W} &= \frac{(\mathbb{U}(0,\hat{\eta}))^2}{n\left(\mathbb{I}_{\beta\beta}(0,\hat{\eta}) - \mathbb{I}_{\beta\eta}(0,\hat{\eta})\mathbb{I}_{\eta\eta}(0,\hat{\eta})^{-1}\mathbb{I}_{\beta\eta}(0,\hat{\eta})^T\right)} \\
&= \frac{\left(\sum_{i=1}^{n} S_\beta(\mathbf{y}_i, 0, \hat{\eta})\right)^2}{n\left(\mathbb{I}_{\beta\beta}(0,\hat{\eta}) - \mathbb{I}_{\beta\eta}(0,\hat{\eta})\mathbb{I}_{\eta\eta}(0,\hat{\eta})^{-1}\mathbb{I}_{\beta\eta}(0,\hat{\eta})^T\right)}
\end{aligned}$$

Estimation of variance

Since there is no closed form for the expected values $\mathbb{I}_{\beta\beta,i}(\beta,\eta)$, $\mathbb{I}_{\beta\eta,i}(\beta,\eta)$, and $\mathbb{I}_{\eta\eta,i}(\beta,\eta)$, we must estimate the variance of the score statistic. Define

$$\bar{\mathcal{I}}_{\beta\eta}(\beta,\eta) = \frac{1}{n}\sum_{i=1}^{n} \mathcal{I}_{\beta\eta}(\mathbf{y}_i, \beta, \eta),$$

and note that as $n \to \infty$, $\bar{\mathcal{I}}_{\beta\eta}(\beta,\eta) \xrightarrow{p} \mathbb{I}_{\beta,\eta}(\beta,\eta)$ (similarly for $\bar{\mathcal{I}}_{\beta\beta}(\beta,\eta)$ and $\bar{\mathcal{I}}_{\eta\eta}(\beta,\eta)$) [3]. We therefore define

$$\mathcal{W} = \frac{\left(\sum_{i=1}^{n} S_\beta(0,\hat{\eta})\right)^2}{n\left(\bar{\mathcal{I}}_{\beta\beta}(0,\hat{\eta}) - \bar{\mathcal{I}}_{\beta\eta}(0,\hat{\eta})\bar{\mathcal{I}}_{\eta\eta}(0,\hat{\eta})^{-1}\bar{\mathcal{I}}_{\beta\eta}(0,\hat{\eta})^T\right)},$$

and refer to the denominator as the asymptotic variance of the efficient score statistic.

Alternatively, we can define

$$\mathcal{U}(\mathbf{y}_i, \beta, \eta) = S_\beta(\mathbf{y}_i, \beta, \eta) - \bar{\mathcal{I}}_{\beta\eta}(\beta,\eta)\bar{\mathcal{I}}_{\eta\eta}(\beta,\eta)^{-1} S_\eta(\mathbf{y}_i, \beta, \eta),$$

and compute the efficient score statistic based on the empirical variance,

$$W = \frac{\left(\sum_{i=1}^{n} S_\beta(\mathbf{y}_i, 0, \hat{\eta})\right)^2}{\sum_{i=1}^{n} \mathcal{U}(\mathbf{y}_i, 0, \hat{\eta})^2}.$$

Testing shows the asymptotic variance and empirical variance to be numerically equivalent (for large enough n). For numerical efficiency, the `groupedSurv` package implements the empirical variance estimation version of the efficient score statistic, *i.e.,* W.

Incorporating family structure

We now expand the above approach (in the absence of covariates) to account for possible correlations in outcome among members of the same family. The genotypic hazard for individual i, within family f of size s_f, is given by equation (1) from [4]:

$$\lambda_i(t|x_i) = \lambda_0(t)e^{\beta x_i + b_i}.$$

Here $\mathbf{b} \in \mathbb{R}^{s_f}$ is a random effect vector distributed according to $N_{s_f}(0, \sigma^2\kappa)$, where the elements of the kinship matrix, κ, are defined as

$$\kappa_{ij} = \begin{cases} 1 & \text{if } i = j \\ 0 & \text{if individuals } i \text{ and } j \text{ are unrelated, (\textit{i.e.,} parent/parent)} \\ \frac{1}{2} & \text{otherwise (parent/offspring or offspring/offspring)} \end{cases}.$$

Without loss of generality, we can reorder our data to group the individuals by family and role within family, *e.g.,* for data consisting of m trios, $\{O_1, F_1, M_1, \ldots, O_m, F_m, M_m\}$. In this case, the kinship matrix for each family, κ_f, would have an identical structure,

$$\kappa_f = \begin{bmatrix} 1 & \frac{1}{2} & \frac{1}{2} \\ \frac{1}{2} & 1 & 0 \\ \frac{1}{2} & 0 & 1 \end{bmatrix}.$$

Corresponding matrices can be constructed for different family compositions, *e.g.,* offspring-parent, offspring-offspring-parent, or offspring-offspring-parent-parent.

Log-likelihood

If conditionally on $\mathbf{b}$ the censoring is independent and non-informative also of $\mathbf{b}$, then, following equation (2) from [4], the likelihood for family f is given by

$$L_f(\beta, \eta) = \int \prod_{i \in \text{Family}_f} \left[\left(1 - \alpha_{k_i}^{\exp(\beta x_i + b_{fi})}\right)^{\delta_i} \prod_{j=1}^{k_i - 1} \alpha_j^{\exp(\beta x_i + b_{fi})} \right] \Phi_{s_f}(\mathbf{b}_f, 0, \sigma^2\kappa_f)\, \mathrm{d}\mathbf{b}_f.$$

Assuming independence across families, and the full log-likelihood is given by

$$l(\beta, \eta) = \sum_{f=1}^{m} \log \left(\int \prod_{i \in \text{Family}_f} \left[\left(1 - \alpha_{k_i}^{\exp(\beta x_i + b_{fi})}\right)^{\delta_i} \prod_{j=1}^{k_i - 1} \alpha_j^{\exp(\beta x_i + b_{fi})} \right] \Phi_{s_f}(\mathbf{b}_f, 0, \sigma^2 \kappa_f) \, \mathrm{d}\mathbf{b}_f \right).$$

Score functions

Using logarithmic differentiation we find,

$$S_\beta(\beta, \eta) = \sum_{f=1}^{m} \frac{1}{L_f(\beta, \eta)} \int \prod_{i \in \text{Family}_f} \left(L(\mathbf{y}_i, \beta, \eta)\right) \sum_{i \in \text{Family}_f} x_i \left[\frac{\delta_i \log(\alpha_{k_i}) \exp(\beta x_i + b_{fi}) \alpha_{k_i}^{\exp(\beta x_i + b_{fi})}}{\alpha_{k_i}^{\exp(\beta x_i + b_{fi})} - 1} \right.$$
$$\left. + \sum_{j=1}^{k_i - 1} \log(\alpha_j) \exp(\beta x_i + b_{fi}) \right] \Phi_{s_f}(\mathbf{b}_f, 0, \sigma^2 \kappa_f) \, \mathrm{d}\mathbf{b}_f,$$

where $L(\mathbf{y}_i, \beta, \eta) = \left(1 - \alpha_{k_i}^{\exp(\beta x_i + b_{fi})}\right)^{\delta_i} \prod_{j=1}^{k_i - 1} \alpha_j^{\exp(\beta x_i + b_{fi})}$.

Similarly,

$$S_{\alpha_j}(\beta, \eta) = \sum_{f=1}^{m} \frac{1}{L_f(\beta, \eta)} \int \prod_{i \in \text{Family}_f} \left(L(\mathbf{y}_i, \beta, \eta)\right) \sum_{i \in \text{Family}_f} \left[\mathbb{1}[k_i = j] \frac{\delta_i \exp(\beta x_i + b_{fi}) \alpha_j^{\exp(\beta x_i + b_{fi}) - 1}}{\alpha_j^{\exp(\beta x_i + b_{fi})} - 1} \right.$$
$$\left. + \mathbb{1}[k_i > j] \frac{\exp(\beta x_i + b_{fi})}{\alpha_j} \right] \Phi_{s_f}(\mathbf{b}_f, 0, \sigma^2 \kappa_f) \, \mathrm{d}\mathbf{b}_f,$$

and

$$S_{\sigma^2}(\beta, \eta) = \sum_{f=1}^{m} \frac{1}{L_f(\beta, \eta)} \int \prod_{i \in \text{Family}_f} \left(L(\mathbf{y}_i, \beta, \eta)\right) \Phi_{s_f}(\mathbf{b}_f, 0, \sigma^2 \kappa_f) \left(\frac{\mathbf{b}_f^T \kappa_f^{-1} \mathbf{b}_f - s_f \sigma^2}{2(\sigma^2)^2} \right) d\mathbf{b}_f.$$

The efficient score statistic is then calculated as stated previously.

Gene- and pathway-level statistics

Within the context of GWAS, what is often of interest is to conduct the analysis at the level of a gene or pathway rather than individual variants. A variety of methods are available for aggregating variant-level statistics [5]. The `groupedSurv` package offers support for such set-based analyses by giving users the option of returning the contribution of sample i to the score statistic for each variant tested. These patient-by-variant-level results can then be used as inputs in aggregate statistics. Alternatively, the included `geneStat()` function accepts a user-specified `R` function as an argument, returning the desired aggregate statistic directly.

Some aggregate statistics incorporate a vector of constants used to up- or down-weight individual variants within the gene- or pathway-level statistics. The structure of the `geneStat()`

argument `geneSet`, used to specify the variants composing the sets to be tested, also allows for specifying weights to be used for each variant, if desired. For more information about the arguments and use of `geneStat()`, please see the associated function documentation or `groupedSurv` vignette within the package.

In the absence of a user-specified aggregate statistic, `geneStat()` implements a Sequence Kernel Association Test (SKAT) [6, 7] type statistic by default. The SKAT statistic is simply a weighted sum of squares of the single variant statistics,

$$S_{\text{SKAT}} = \sum_{j=1}^{m} \omega_j^2 W_j^2.$$

The associated set-level *P*-values can then be computed either from a mixed chi-square distribution, or through permutation.

Coding CALGB 40101 grouped survival data

One way to understand how grouped survival data should be coded for `groupedSurv` is to consider it within the context of the likelihood function.

$$L_i(\mathbf{y}_i, \beta, \eta) = (1 - \alpha_{k_i}^{\exp(\mathbf{x}_i^T\beta + \mathbf{z}_i^T\theta)})^{\delta_i} \prod_{j=1}^{k_i-1} \alpha_j^{\exp(\mathbf{x}_i^T\beta + \mathbf{z}_i^T\theta)}$$

The first term, which is raised to the power of δ_i, represents the probability of failing in interval k_i. The second term is the product of the probabilities of surviving the entirety of the previous intervals, 1 to $k_i - 1$. The contribution of each patient to the total likelihood then is composed of the combination of the intervals they survived without incident, and that in which the event occurred, if applicable.

In CALGB 40101, the event of interest is the minimal dose needed to cause paclitaxel-induced grade 2 or higher peripheral neuropathy. Patients receive up to six cycles of paclitaxel, meaning the continuous interval of all possible doses of paclitaxel, $[0, \infty)$ is divided by the six doses into $r = 7$ intervals: $[0, 1), [1, 2), [2, 3), [3, 4), [4, 5), [5, 6), [6, \infty)$. The coding of $\delta_i = 0$ or 1 should be independent of the treatment arm to which a patient was randomized. Given that there are only seven intervals, and only two event states, it is possible to enumerate all possible codings, which we do in Tables S1 and S2.

In the case of CALGB 40101, special attention must be given to distinguish the interval numbers used in the likelihood equation, $k_i \in \{1, 2, 3, 4, 5, 6, 7\}$, and the dosages taken as arguments in `groupedSurv`, `gtime` $\in \{$*1 cycle, 2 cycles, 3 cycles, 4 cycles, 5 cycles, 6 cycles,* $\infty\}$.

Table S1 considers the scenarios in which a patient is observed to have had the event. Note that there should be no events observed prior to the first cycle. In the case that an event is not observed for a particular patient, that patient will be censored, (Table S2). Note that patients who drop out prior to the first cycle contribute no information to the likelihood.

Table S1: Coding patients with observed events.

Scenario	Interpretation	k_i	δ_i	`gtime`
Event after 1 cycle	Minimal dose < 1, *i.e.,* failed in interval 1	1	1	1
Event after 2 cycles	$1 <$ Minimal dose < 2, *i.e.,* survived interval 1, failed in interval 2	2	1	2
Event after 3 cycles	$2 <$ Minimal dose < 3, *i.e.,* survived intervals 1-2, failed in interval 3	3	1	3
Event after 4 cycles	$3 <$ Minimal dose < 4, *i.e.,* survived intervals 1-3, failed in interval 4	4	1	4
Event after 5 cycles	$4 <$ Minimal dose < 5, *i.e.,* survived intervals 1-4, failed in interval 5	5	1	5
Event after 6 cycles	$5 <$ Minimal dose < 6, *i.e.,* survived intervals 1-5, failed in interval 6	6	1	6

Table S2: Coding patients with no observed events.

Scenario	Interpretation	k_i	δ_i	`gtime`
Drops out before cycle 1	Minimal dose unknown, *i.e.,* survives zero intervals	1	0	1
Receives 1 cycle, no event, drops out before cycle 2	Minimal dose > 1, *i.e.,* survives interval 1	2	0	2
Receives 2 cycles, no event, drops out before cycle 3	Minimal dose > 2, *i.e.,* survives intervals 1-2	3	0	3
Receives 3 cycles, no event, drops out before cycle 4	Minimal dose > 3, *i.e.,* survives intervals 1-3	4	0	4
Receives 4 cycles, no event, drops out before cycle 5	Minimal dose > 4, *i.e.,* survives intervals 1-4	5	0	5
Receives 5 cycles, no event, drops out before cycle 6	Minimal dose > 5, *i.e.,* survives intervals 1-5	6	0	6
Receives 6 cycles, no event prior to study close	Minimal dose > 6, *i.e.,* survives intervals 1-6	7	0	∞

Simulation

Grouped survival data are generated by first simulating continuous right-censored survival times, and then converting these times to grouped failure times. For each of n patients, a parameter of interest, x, is simulated from a binomial distribution of size 2 and probability of success equal to a specified minor allele frequency (MAF), representing a SNP under the additive genetic model. For all simulations, two baseline covariates, z_1 and z_2, are simulated from a standard normal distribution and a Bernoulli distribution with probability of success equal to 0.5, respectively. The values of z_2 are centered by subtracting 0.5. Continuous survival times are then simulated from an exponential model with hazard

$$\lambda(t|\mathbf{x}_i, \mathbf{z}_i) = \lambda_0(t) \exp(\mathbf{x}_i^T \beta + \mathbf{z}_i^T \theta),$$

Continuous censoring times are independently generated from a uniform distribution over the interval $(0, c_{\max})$. We then specify grouped survival times, representing fixed observation times in a hypothetical study, by first selecting a maximum observed time, τ, and then dividing the interval $[0, \tau)$ into five equal subintervals. Grouped failure times and event indicators are then assigned as described in the main article.

Table S3 gives the parameter values for the different simulations. In all cases, a baseline hazard of $\lambda_0 = 1$ and nuisance parameters of $\theta_1 = 0.2$ and $\theta_2 = 0.2$ are used. The $c_{\max}$ and τ parameters are adjusted based on the MAF so as to generate empirical event rates of 60%. All reported results are based on $B = 10,000$ replicates of each set of simulation parameters, except for the comparison of performance using different numbers of CPU cores, where the results are the average of ten replicates.

Results

Empirical bias assessments for `groupedSurv` and the `coxph()` function from the `survival` package (using the exact likelihood method to adjust for ties) are shown in Figure S1. An event rate of 0.6 and minor allele frequency of 0.5 is used for each of $B = 10,000$ simulation replicates for sample sizes of $n = 500$, $1,000$, and $3,000$. The simulations were conducted under the null hypothesis ($\beta = 0$). Our approach produces evidently unbiased estimates regardless of sample size, while the exact likelihood method for right-censored data seemingly underestimates the effect size for all but the largest sample size simulated.

Figure S1: Effect Size Estimation Bias for `groupedSurv` and `coxph()`. Box plot comparing bias of effect size estimation of `groupedSurv` and `coxph()`.

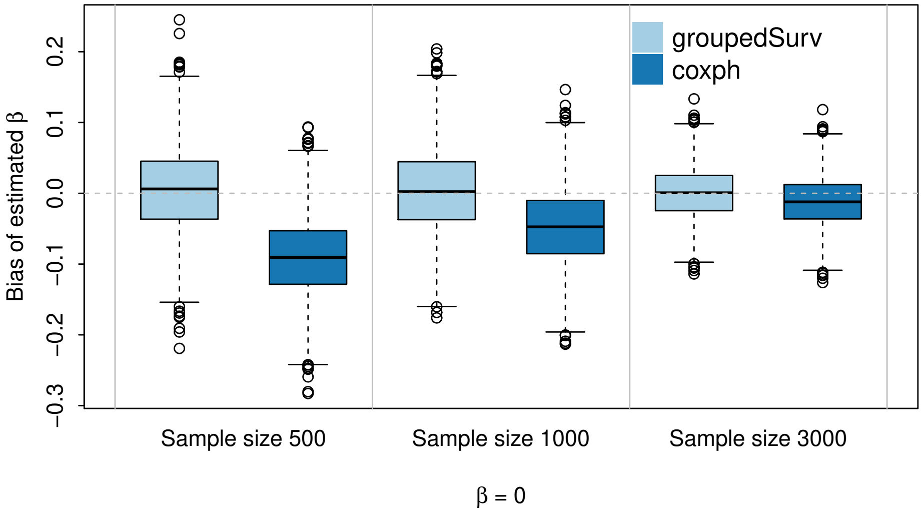

Empirical assessment of the type I error for `groupedSurv` for a sample size of $n = 500$ are shown in Figure S2. The type I error simulation results for allele frequencies of 0.05, 0.2 and 0.5, and event rates of 0.3, 0.5 and 0.7, are shown, with $B = 10,000$ simulation replicates used for each example.

Figure S2: Box Plot for Type I Error for `groupedSurv`. Box plot of type 1 error of `groupedSurv` for different event rates and minor allele frequencies, with a sample size of $n = 500$.

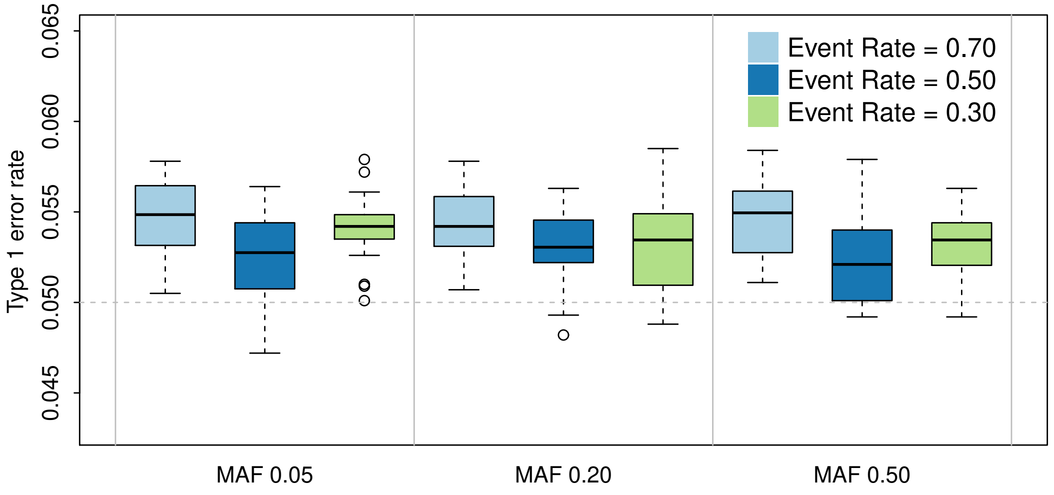

Simulation results of power estimations for the grouped failure time method for a sample size of $n = 500$ are shown in Figure S3. Minor allele frequencies of 0.05, 0.1, 0.2 and 0.5, and an event rate of 0.6 are used for each example, with the effect size varying over the range of $\beta \in (-0.9, 0.9)$. Power is estimated at the two-sided $\alpha = 0.05$ level using $B = 10,000$ replicates.

Figure S3: Power for `groupedSurv`. Power estimates for `groupedSurv` for different minor allele frequencies, with a sample size of $n = 500$.

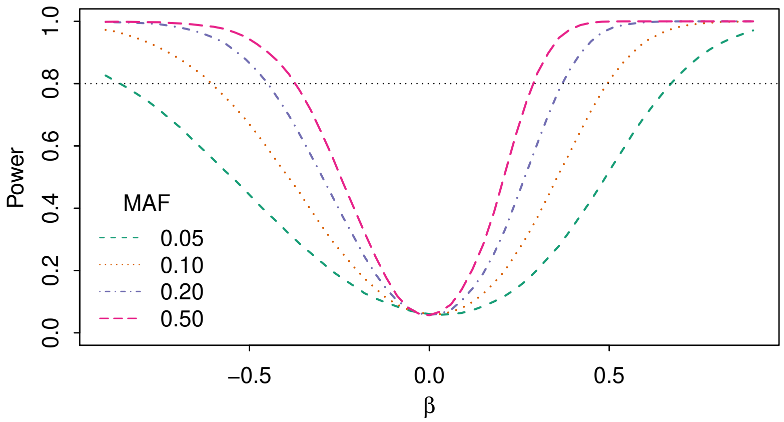

Figure S4 provides non-parametric maximum likelihood estimates of neuropathy-free survival by genotype, and the corresponding LocusZoom [8] plots, for six SNPs highlighted in the Discussion section of the main article: `rs9435869` (variant in intergenic region of *RHOU* and *RAB4A*; *P*-value $< 2.5 \times 10^{-6}$, MAF $= 0.15$, $\hat{\beta} = -0.67$), `rs9684260` (variant in intergenic region of *LINC02232* and *EPHA5*; *P*-value $< 5.1 \times 10^{-6}$, MAF $= 0.36$, $\hat{\beta} = 0.48$), `rs6481837`

(variant in intergenic region of *ITGB1* and *NRP1*; *P*-value $< 6.3 \times 10^{-6}$, MAF $= 0.39$, $\hat{\beta} = -0.45$), `rs11680024` (variant in intergenic region of *FSHR* and *NRXN1*; *P*-value $< 1.5 \times 10^{-5}$, MAF $= 0.12$, $\hat{\beta} = -0.71$), `rs2233335` (variant in intron region of *NDRG1*; *P*-value $< 3.7 \times 10^{-5}$, MAF $= 0.38$, $\hat{\beta} = -0.44$), and `rs10891842` (variant in intron region of *CAMD1*; *P*-value $< 4.0 \times 10^{-5}$, MAF $= 0.42$, $\hat{\beta} = 0.4$).

References

[1] Prentice, R.L., Gloeckler, L.A.: Regression analysis of grouped survival data with application to breast cancer data. Biometrics **34**(1), 57–67 (1978)

[2] Tsiatis, A.A.: Semiparametric Theory and Missing Data. Springer, New York, NY (2006)

[3] Freedman, D.A.: How can the score test be inconsistent? The American Statistician **61**(4), 291–295 (2007). doi:10.1198/000313007X243061. https://doi.org/10.1198/000313007X243061

[4] Ripatti, S., Palmgren, J.: Estimation of multivariate frailty models using penalized partial likelihood. Biometrics **56**(4), 1016–1022 (2004). doi:10.1111/j.0006-341X.2000.01016.x. https://onlinelibrary.wiley.com/doi/pdf/10.1111/j.0006-341X.2000.01016.x

[5] Holden, M., Deng, S., Wojnowski, L., Kulle, B.: GSEA-SNP: applying gene set enrichment analysis to SNP data from genome-wide association studies. Bioinformatics **24**(23), 2784–2785 (2008). doi:10.1093/bioinformatics/btn516

[6] Wu, M.C., Lee, S., Cai, T., Li, Y., Boehnke, M., Lin, X.: Rare-variant association testing for sequencing data with the sequence kernel association test. American Journal of Human Genetics **89**(1), 82–93 (2011)

[7] Ionita-Laza, I., Lee, S., Makarov, V., Buxbaum, J.D., Lin, X.: Sequence kernel association tests for the combined effect of rare and common variants. American Journal of Human Genetics **92**(6), 841–53 (2013). doi:10.1016/j.ajhg.2011.05.029

[8] Pruim, R.J., Welch, R.P., Sanna, S., Teslovich, T.M., Chines, P.S., Gliedt, T.P., Boehnke, M., Abecasis, G.R., Willer, C.J.: LocusZoom: regional visualization of genome-wide association scan results. Bioinformatics **26**(18), 2336–2337 (2010). doi:10.1093/bioinformatics/btq419

Table S3: Summary of parameters for each simulation

Simulations				
	Sample size	Number of SNPs	β	MAF
Efficient score type I error	1×10^3	1×10^6	0	0.05
	1×10^3	1×10^6	0	0.20
	1×10^3	1×10^6	0	0.50
	1×10^3	1×10^6	0	0.05
	1×10^3	1×10^6	0	0.20
	1×10^3	1×10^6	0	0.50
	1×10^3	1×10^6	0	0.05
	1×10^3	1×10^6	0	0.20
	1×10^3	1×10^6	0	0.50
Bias estimation	1×10^3	1×10^6	0, 1	0.05
	1×10^3	1×10^6	0, 1	0.20
	1×10^3	1×10^6	0, 1	0.50
Power calculation	1×10^3	1×10^6	[-0.9, 0.9]	0.05
	1×10^3	1×10^6	[-0.9, 0.9]	0.10
	1×10^3	1×10^6	[-0.9, 0.9]	0.20
	1×10^3	1×10^6	[-0.9, 0.9]	0.50
Timing benchmark	2×10^2	2×10^5	0	0.50
	5×10^2	2×10^5	0	0.50
	1×10^3	2×10^5	0	0.50
	2×10^2	6×10^5	0	0.50
	5×10^2	6×10^5	0	0.50
	1×10^3	6×10^5	0	0.50
	2×10^2	1×10^6	0	0.50
	5×10^2	1×10^6	0	0.50
	1×10^3	1×10^6	0	0.50
Supplementary Information Simulations				
	Sample size	Number of SNPs	β	MAF
Bias estimation	5×10^2	1×10^6	0, 1	0.50
	1×10^3	1×10^6	0, 1	0.50
	3×10^3	1×10^6	0, 1	0.50
Efficient score type I error	5×10^2	1×10^6	0	0.05
	5×10^2	1×10^6	0	0.20
	5×10^2	1×10^6	0	0.50
	5×10^2	1×10^6	0	0.05
	5×10^2	1×10^6	0	0.20
	5×10^2	1×10^6	0	0.50
	5×10^2	1×10^6	0	0.05
	5×10^2	1×10^6	0	0.20
	5×10^2	1×10^6	0	0.50
Power calculation	5×10^2	1×10^6	[-0.9, 0.9]	0.05
	5×10^2	1×10^6	[-0.9, 0.9]	0.10
	5×10^2	1×10^6	[-0.9, 0.9]	0.20
	5×10^2	1×10^6	[-0.9, 0.9]	0.50

Figure S4: Non-parametric maximum likelihood survival function estimates and regional visualization plots. Survival and LocusZoom [8] plots for six selected SNPs.

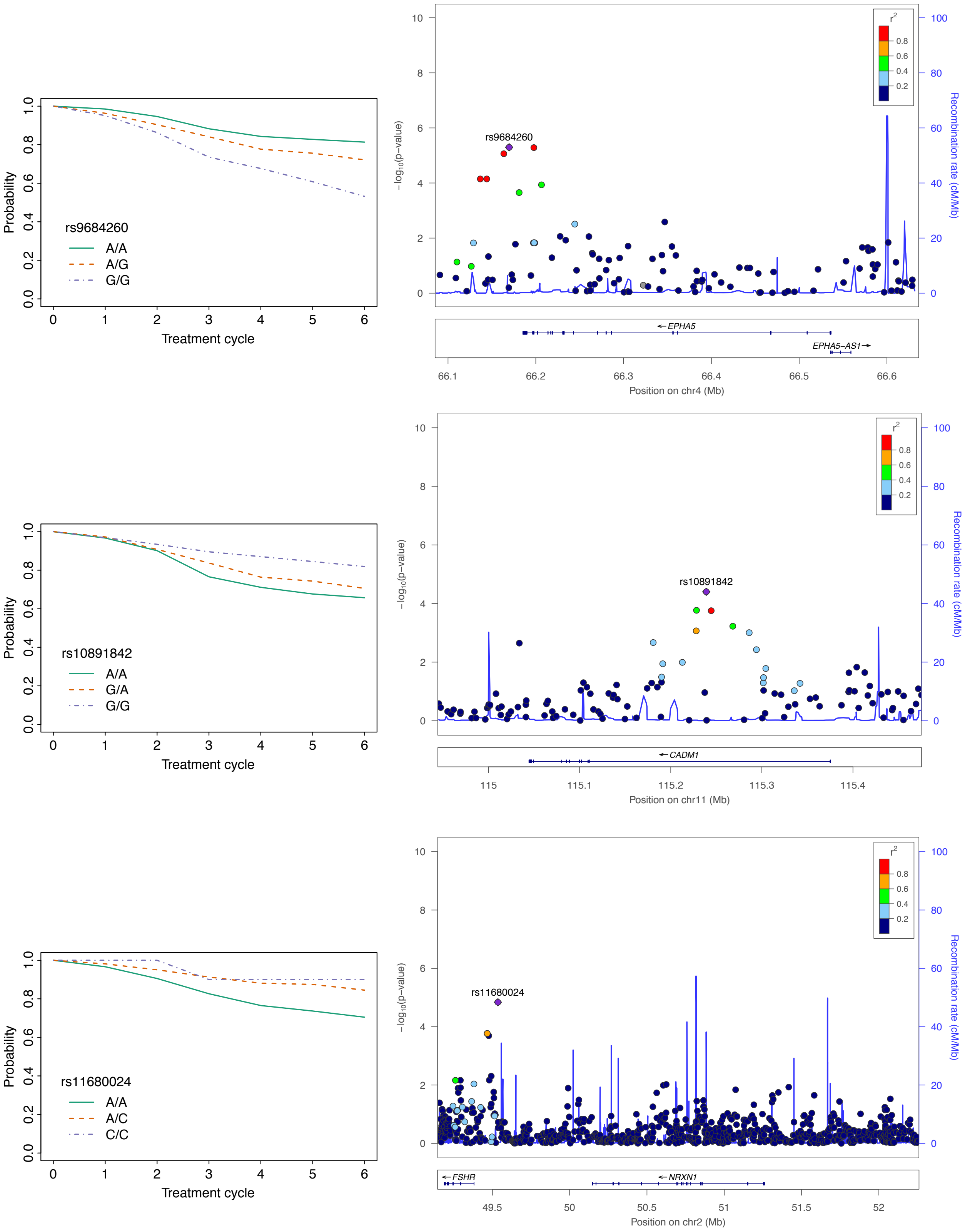

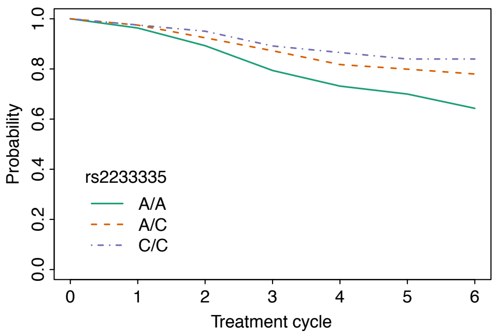
Probability
Treatment cycle
rs2233335
A/A
A/C
C/C

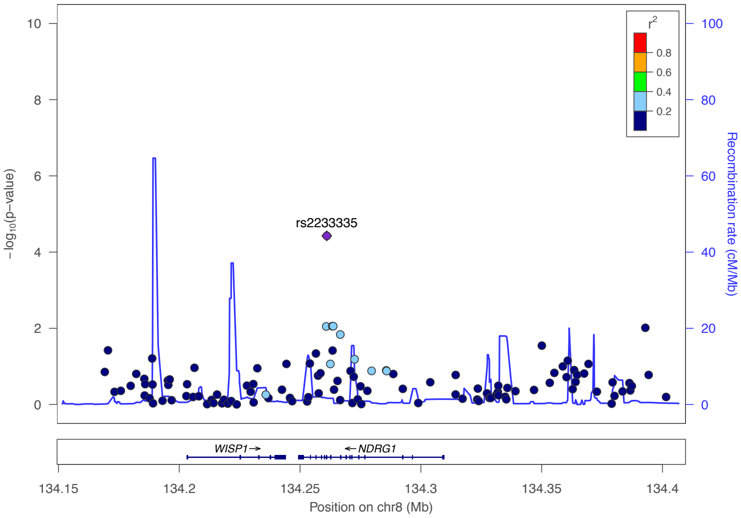
−log10(p−value)
Recombination rate (cM/Mb)
r2
rs2233335
WISP1→
←NDRG1
Position on chr8 (Mb)

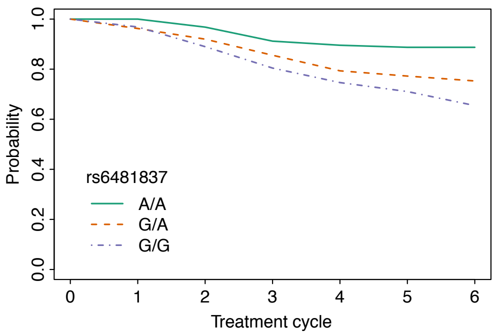
Probability
Treatment cycle
rs6481837
A/A
G/A
G/G

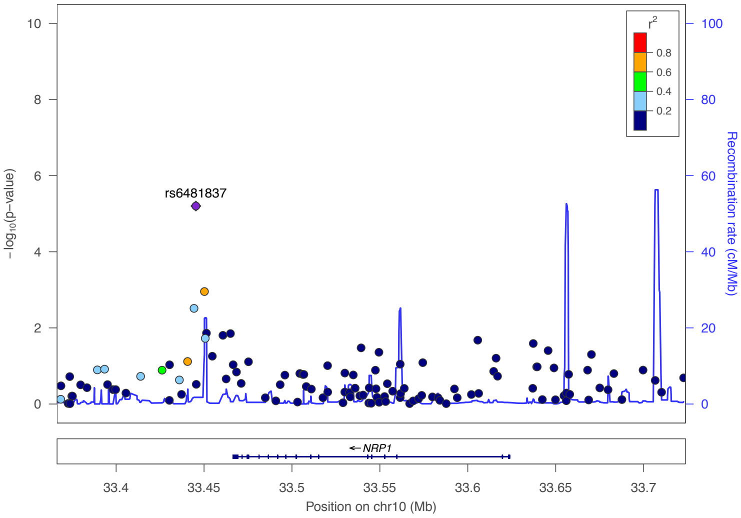
−log10(p−value)
Recombination rate (cM/Mb)
r2
rs6481837
←NRP1
Position on chr10 (Mb)

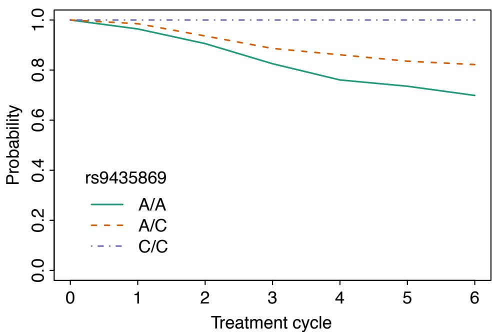
Probability
Treatment cycle
rs9435869
A/A
A/C
C/C

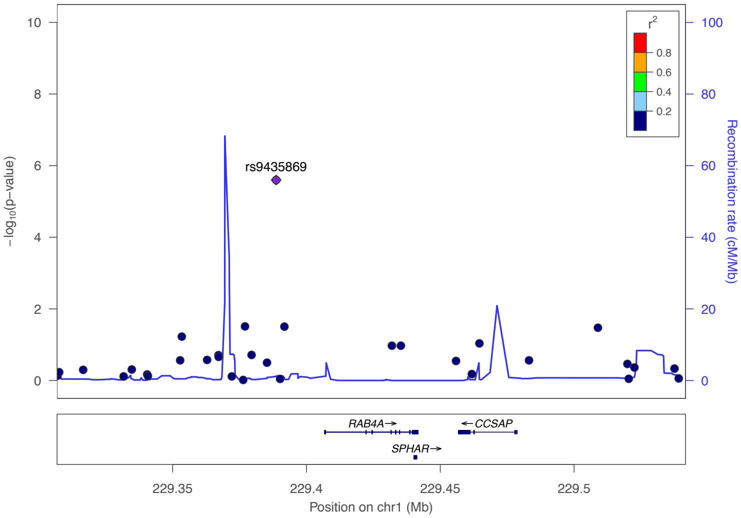
−log10(p−value)
Recombination rate (cM/Mb)
r2
rs9435869
RAB4A→
←CCSAP
SPHAR→
Position on chr1 (Mb)
