# Supplementary material for: Efficient estimation of grouped survival models
Source: BMC Bioinformatics. 2019 May 28;20:269. doi: 10.1186/s12859-019-2899-x (PMC6540566; doi:10.1186/s12859-019-2899-x)
Supplement: Supplementary file 2 — knitr-generated slides showing R code used to reproducibly conduct the data simulations, summarize the operating characteristics, and estimate the processing benchmarks. (PDF 112 kb) [file 12859_2019_2899_MOESM2_ESM.pdf]

Simulation and Benchemark for groupedSurv

By:Jiaxing Lin and Alex Sibley

June 8, 2018

Loading R packages and Initial Setting up

```
library(foreach)
library(doRNG)
library(doParallel)
library(survival)
library(groupedSurv)
library(microbenchmark)
ncores <- 48
registerDoParallel(cores = ncores)
set.seed(545)
```

Data Simulation Function

```r
simUnifCens <- function(n = 500, cmax = 2, lam0 = 1, maxbreakq = 0.9, ntps = 5,
  beta = 1, theta = c(0.2, 0.2), MAF = 0.5) {
  r <- ntps + 1
  maxbreak <- qexp(maxbreakq, lam0)
  x1 <- rnorm(n); x2 <- rbinom(n, 1, 0.5) - 0.5
  g <- matrix(rbinom(n, 2, MAF), ncol = 1)
  xmat <- matrix(cbind(x1, x2), ncol = 2)
  lami <- lam0 * exp(g * beta + xmat %*% theta)
  stime <- rexp(n, lami); ctime <- runif(n, 0, cmax)
  event <- stime < ctime; otime <- pmin(stime, ctime)
  breaks <- (1:ntps) * (maxbreak/ntps)
  gtime <- findInterval(otime, breaks) + 1
  event[gtime == r] <- FALSE
  dctime <- findInterval(ctime, breaks) + 1
  event[gtime == dctime] <- FALSE
  gtime[which(gtime==r)] <- Inf
  N <- colSums(outer(gtime, 0.9:(ntps - 0.1), FUN = ">"))
  D <- as.vector(table(gtime[event]))
  alphaHat <- (N - D)/N
  gammaHat <- log(-log(alphaHat))
  start <- c(0, breaks[-ntps])
  end <- breaks
  alpha <- exp(lam0 * (start - end))
  gamma <- log(-log(alpha))
        return(list(X = xmat, gtime = gtime, delta = event, rate = sum(event)/n, ntps = ntps,
              g = g, x1 = x1, x2 = x2, alphaHat = alphaHat, beta = beta, theta = theta,
              gammaHat = gammaHat, alpha = alpha, gamma = gamma ))
}
```

Bias Simulation Function

```r
biasEst <- function(n, erate, ntps = 5, B = 500, cores = ncores, maf = 0.5, beta = 1, maxq = 0.7) {
   reps <- foreach(b= 1:B, .combine = rbind) %dorng% {
        simdat <- simUnifCens(n = n, cmax = erate, lam0 = 1, maxbreakq = maxq,
          ntps = ntps, MAF = maf, beta = 1)  ## erate is used as cmax
        ThetaHat <- c(simdat$gammaHat, rep(0, length(simdat$theta)))
        simdat$X <- cbind(simdat$g, simdat$X)
        Est <- thetaEst(simdat$X, simdat$gtime, simdat$delta)
        simdat$gtime[which(simdat$gtime==Inf)] <- ntps+1
        coxphResEfron <- coxph(Surv(gtime, delta) ~ g + x1 + x2, data = simdat, ties = "efron")$coefficients
        coxphResExact <- coxph(Surv(gtime, delta) ~ g + x1 + x2, data = simdat, ties = "exact")$coefficients
        Bias <- c(Est$alpha, Est$theta) - c(simdat$alpha, simdat$beta, simdat$theta)
        coxphEfronBias <- coxphResEfron - c(simdat$beta, simdat$theta)
        coxphExactBias <- coxphResExact - c(simdat$beta, simdat$theta)
        return(c(simdat$rate, Bias, coxphEfronBias, coxphExactBias))
    }
    colnames(reps) <- c("EventRate", paste0("gamma", 1:ntps), "beta", paste0("theta", 1:2),
      "efron beta", paste0("theta", 1:2), "exact beta", paste0("theta", 1:2))
          means <- colMeans(reps)
    rate <- round(means[1], 2)
    return(list(reps,rate))
}
```

Bias Simulation

```
res_05 <- biasEst(n = 1000, erate = 4, beta = 1, maf = 0.05, maxq = 0.67)
res_20 <- biasEst(n = 1000, erate = 4, beta = 1, maf = 0.2, maxq = 0.54)
res_50 <- biasEst(n = 1000, erate = 4, beta = 1, maf = 0.5, maxq = 0.31)
res_05_beta_0 <- biasEst(n = 1000, erate = 4, beta = 0, maf = 0.05, maxq = 0.67)
res_20_beta_0 <- biasEst(n = 1000, erate = 4, beta = 0, maf = 0.2, maxq = 0.54)
res_50_beta_0 <- biasEst(n = 1000, erate = 4, beta = 0, maf = 0.5, maxq = 0.31)
```

```
bias_maf_05_beta_1 <- res_05[[1]][, c(7, 10)]
bias_maf_05_beta_1_exact <- res_05[[1]][, 13]
bias_maf_20_beta_1 <- res_20[[1]][, c(7, 10)]
bias_maf_20_beta_1_exact <- res_20[[1]][, 13]
bias_maf_50_beta_1 <- res_50[[1]][, c(7, 10)]
bias_maf_50_beta_1_exact <- res_50[[1]][, 13]
bias_maf_05_beta_0 <- res_05_beta_0[[1]][, c(7, 10)]
bias_maf_05_beta_0_exact <- res_05_beta_0[[1]][, 13]
bias_maf_20_beta_0 <- res_20_beta_0[[1]][, c(7, 10)]
bias_maf_20_beta_0_exact <- res_20_beta_0[[1]][, 13]
bias_maf_50_beta_0 <- res_50_beta_0[[1]][, c(7, 10)]
bias_maf_50_beta_0_exact <- res_50_beta_0[[1]][, 13]
save(bias_maf_50_beta_1, bias_maf_20_beta_1, bias_maf_05_beta_1,
       bias_maf_50_beta_0, bias_maf_20_beta_0, bias_maf_05_beta_0,
       bias_maf_50_beta_1_exact, bias_maf_20_beta_1_exact, bias_maf_05_beta_1_exact,
       bias_maf_50_beta_0_exact, bias_maf_20_beta_0_exact, bias_maf_05_beta_0_exact,
       file = "../../Result/biasBetaSim_test.RData")
```

Extra Bias Simulation

```
SI_Bias_Size_500 <- biasEst(n = 500, erate = 4, beta = 1, maf = 0.5, maxq = 0.67)
SI_Bias_500 <- SI_Bias_Size_500[[1]][, c(7, 10)]
SI_Bias_Size_1000 <- biasEst(n = 1000, erate = 4, beta = 1, maf = 0.5, maxq = 0.54)
SI_Bias_1000 <- SI_Bias_Size_1000[[1]][, c(7, 10)]
SI_Bias_Size_3000 <- biasEst(n = 3000, erate = 4, beta = 1, maf = 0.5, maxq = 0.31)
SI_Bias_3000 <- SI_Bias_Size_3000[[1]][, c(7, 10)]
save(SI_Bias_Size_500,  SI_Bias_500,  eventRate_500,
     SI_Bias_Size_1000, SI_Bias_1000, eventRate_1000,
     SI_Bias_Size_3000, SI_Bias_3000, eventRate_3000,
                 file = "../../Result/SI_biasBeta_Size.RData")
```

Statistical Characterstics

```r
Replicate <- function(n, rate, ntps = 5, B = 10000, maf = 0.05, beta = 0, maxq = 0.7) {
  reps <- foreach(b = 1:B, .combine = rbind) %dorng% {
    simdat <- simUnifCens(n = n, cmax = rate, lam0 = 1, maxbreakq = maxq, ntps = ntps,
                          MAF = maf, beta = beta)
    thetaest <- thetaEst(simdat$X, simdat$gtime, simdat$delta)
    res <- groupedSurv(x = simdat$g, Z = simdat$X, alpha = thetaest$alpha, theta = thetaest$theta,
                       gtime = simdat$gtime, delta = simdat$delta, beta = 0, nCores = 1)
    return(c(res, simdat$rate))
  }
  reps
}
```

Type One Error

```
t1etest <- function(n, rate, ntps = 5, B = 10000, maf = 0.05, beta = 0, maxq = 0.7)
{
        reps <- Replicate(n, rate, ntps, B, maf, beta, maxq)
        t1e <- sum(reps[, 2] <= 0.05)/B
   erate <- round(mean(c(unlist(reps[, 3]))), 2)
   return(cbind(t1e, erate))
}
B <- 500
res_maf_05_erate_70 <- NULL
res_maf_20_erate_70 <- NULL
res_maf_50_erate_70 <- NULL
for (i in 1:20) {
  res_maf_05_erate_70[i] <- t1etest(n = 1000, rate = 5, ntps = 5, B = B, maf = 0.05,
    beta = 0, maxq = 0.85)[1]
  res_maf_20_erate_70[i] <- t1etest(n = 1000, rate = 5, ntps = 5, B = B, maf = 0.2,
    beta = 0, maxq = 0.85)[1]
  res_maf_50_erate_70[i] <- t1etest(n = 1000, rate = 5, ntps = 5, B = B, maf = 0.5,
    beta = 0, maxq = 0.85)[1]
}
```

Type One Error

```
res_maf_05_erate_50 <- NULL
res_maf_20_erate_50 <- NULL
res_maf_50_erate_50 <- NULL
for (i in 1:20) {
  res_maf_05_erate_50[i] <- t1etest(n = 1000, rate = 5, ntps = 5, B = B, maf = 0.05,
    beta = 0, maxq = 0.54)[1]
  res_maf_20_erate_50[i] <- t1etest(n = 1000, rate = 5, ntps = 5, B = B, maf = 0.2,
    beta = 0, maxq = 0.54)[1]
  res_maf_50_erate_50[i] <- t1etest(n = 1000, rate = 5, ntps = 5, B = B, maf = 0.5,
    beta = 0, maxq = 0.54)[1]
}
res_maf_05_erate_30 <- NULL
res_maf_20_erate_30 <- NULL
res_maf_50_erate_30 <- NULL
for (i in 1:20) {
  res_maf_05_erate_30[i] <- t1etest(n = 1000, rate = 5, ntps = 5, B = B, maf = 0.05,
    beta = 0, maxq = 0.31)[1]
  res_maf_20_erate_30[i] <- t1etest(n = 1000, rate = 5, ntps = 5, B = B, maf = 0.2,
    beta = 0, maxq = 0.31)[1]
  res_maf_50_erate_30[i] <- t1etest(n = 1000, rate = 5, ntps = 5, B = B, maf = 0.5,
    beta = 0, maxq = 0.31)[1]
}
save(res_maf_05_erate_70, res_maf_20_erate_70, res_maf_50_erate_70,
     res_maf_05_erate_50, res_maf_20_erate_50, res_maf_50_erate_50,
     res_maf_05_erate_30, res_maf_20_erate_30, res_maf_50_erate_30,
     file = "../../Result/T1EScore.Rdata")
```

Performance Benchmark

```
timeBench <- function(n = 1000, m = 1000, rate = 4, ntps = 5, maf = 0.5, beta = 0,
  maxq = 0.31, ncores = 8, repeats = 1, beta=0) {
  g <- matrix(rbinom(n * m, 2, 0.5), ncol = m)
  simdat <- simUnifCens(n = n, cmax = rate, lam0 = 1, maxbreakq = maxq, ntps = ntps,
    MAF = maf, beta = beta)
  thetaest <- thetaEst(simdat$X, simdat$gtime, simdat$delta)
  res <- microbenchmark(groupedSurv(x = g, Z = simdat$X, alpha = thetaest$alpha, theta = thetaest$theta,
    gtime = simdat$gtime, delta = simdat$delta, beta = 0, nCores = ncores),
    times = repeats)
  mean(res$time/(10^9))
}
sizes <- c(200, 500, 1000)
SNPs <- c(2e+05, 6e+05, 1e+06)
timing_Size_SNPs <- matrix(rep(0, length(sizes)*length(SNPs)), ncol = length(SNPs))

i <- 1
for (Size in sizes){
j <- 1
  for (SNP in SNPs) {
    timing_Size_SNPs[i, j] <- timeBench(ncores = 8, m = SNP, n = Size, repeats = 10)
    j <- j + 1
}
i <- i + 1
}
colnames(timing_Size_SNPs) <- paste0("SNPNum ", SNPs )
save(timing_Size_SNPs, file = "../../Result/timging_SNPNum.RData")
```

Performance Benchmark

```
powerCal <- function(beta, n, rate, ntps = 5, B = 100, MAF = 0.5) {
  reps <- foreach(b = 1:B, .combine = rbind) %dorng% {
    simdat <- simUnifCens(n = n, cmax = rate, lam0 = 2.65, maxbreakq = 0.32,
      ntps = ntps, beta = beta, MAF = MAF)  ## rate is used as cmax
    thetaest <- thetaEst(Z = simdat$X, gtime = simdat$gtime, delta = simdat$delta)
    res <- groupedSurv(x = simdat$g, Z = simdat$X, alpha = thetaest$alpha, theta = thetaest$theta,
      gtime = simdat$gtime, delta = simdat$delta, beta = 0, nCores = 1)
    return(c(res))
  }
  power <- mean(c(unlist(reps[, 2])) < 0.05)
  return(data.frame(Beta = beta, Power = power))
}
set.seed(545)
B <- 100
Pow_tmp <- NULL
Pow <- NULL
betaRange <- -30:30 * 0.03
MAFRange <- c(0.05, 0.1, 0.2, 0.5)
for (ns in c(855)) {
  for (rates in c(1)) {
    for (MAFs in MAFRange) {
      i <- 1
      for (betas in betaRange) {
        Pow_tmp[i] <- powerCal(beta = betas, n = ns, rate = rates, ntps = 6, B = B, MAF = MAFs)$Power
        i <- i + 1
      }
      if (is.null(Pow))
        Pow <- Pow_tmp else Pow <- cbind(Pow, Pow_tmp)}}}
colnames(Pow) <- paste0("MAF_", MAFRange)
rownames(Pow) <- paste0("beta_", betaRange)
save(Pow, file = "../../Result/power.RData")
```

Create Figures for Bias

```
load("../../Result/biasBetaSim.RData")
bias_dat_beta_0 <- cbind(bias_maf_05_beta_0, bias_maf_05_beta_0_exact,
                         bias_maf_20_beta_0, bias_maf_20_beta_0_exact,
                         bias_maf_50_beta_0, bias_maf_50_beta_0_exact)
setEPS()
postscript("../../Figure/biasEstBeta.eps", width = 8, height = 8)
par(mfrow = c(2, 1),mgp = c(1.9, 0.6, 0), tcl = -0.2, mar = c(4, 3, 1, 1))
colreal <- rep(brewer.pal(n = 3, "Paired"),3)
ylab <- expression(paste("Bias of estimated ", hat(beta) ))
boxplot(bias_dat_beta_0, ylab = ylab,  sub = expression(paste(beta," = 0")),
        boxwex = 0.6, medlwd = 2, xaxt = "n", col = colreal, ylim=c(-0.5,0.6))
axis(1, at = seq(2, 10, 3), labels = paste("MAF =", c("0.05", "0.20", "0.50")),
     tick = FALSE, cex = 0.3)
for (i in seq(0.5, 20, 3))
  abline(v = i, lty = 1, col = "grey")
abline(h = 0, lty = 2, col = "black")
legend("topright", legend = c("groupedSurv", "coxph efron", "coxph exact"), col = colreal[1:3],
         pch = 15, bty = "n", pt.cex = 3, cex = 1.2, horiz = F, inset = c(0.1, 0.0))
bias_dat_beta_1 <- cbind(bias_maf_05_beta_1, bias_maf_05_beta_1_exact,
                         bias_maf_20_beta_1, bias_maf_20_beta_1_exact,
                         bias_maf_50_beta_1, bias_maf_50_beta_1_exact)
boxplot(bias_dat_beta_1, ylab = ylab,  sub = expression(paste(beta," = 1")),
        boxwex = 0.6, medlwd = 2, xaxt = "n", col = colreal, ylim=c(-0.5,0.6))
axis(1, at = seq(2, 10, 3), labels = paste("MAF =", c("0.05", "0.20", "0.50")),
     tick = FALSE, cex = 0.3)
for (i in seq(0.5, 20, 3))
  abline(v = i, lty = 1, col = "grey")
abline(h = 0, lty = 2, col = "black")
legend("topright", legend = c("groupedSurv", "coxph efron", "coxph exact"), col = colreal[1:3],
       pch = 15, bty = "n", pt.cex = 3, cex = 1.2, horiz = F, inset = c(0.1, 0.0))
dev.off()
```

Create Figure for Type I Error

```
load("../../Result/T1EScore.Rdata")
t1e <- cbind(res_maf_05_erate_70, res_maf_05_erate_50, res_maf_05_erate_30,
             res_maf_20_erate_60, res_maf_20_erate_50, res_maf_20_erate_30,
             res_maf_50_erate_60, res_maf_50_erate_50, res_maf_50_erate_30)
setEPS()
postscript("../../Figure/T1EScore.eps", width = 8, height = 4)
ylab <- "Type 1 error rate"
par(mgp = c(1.9, 0.6, 0), tcl = -0.2, mar = c(3, 3, 1, 1))
colreal <- rep(brewer.pal(n = 3, "Paired"),3)
boxplot(t1e, ylab = ylab, boxwex = 0.6, medlwd = 2, xaxt = "n", col = colreal, ylim=c(.043,0.060))
axis(1, at = seq(2, 10, 3), labels = paste0("MAF ", c("0.05", "0.20", "0.50")),
     tick = FALSE, cex = 0.3)
for (i in seq(0.5, 22, 3))
  abline(v = i, lty = 1, col = "grey")
abline(h = 0.05, lty = 2, col = "gray")
legend("topright", legend = c("Event Rate = 0.70", "Event Rate = 0.50", "Event
Rate = 0.30"), col = colreal[1:3], pch = 15, bty = "n", pt.cex = 3, cex = 1.2,
inset = c(0.04, 0.0))
dev.off()
```

Create Figure for Timing Benchmark

```
load("../../Result/timging.RData")
setEPS()
postscript("../../Figure/Performance.eps", width = 6, height = 6)
par(mfrow = c(2, 1), mgp = c(1.9, 0.6, 0), tcl = -0.2, mar = c(3, 3, 1, 1))
color <-rep(brewer.pal(3, "Accent"),3)
col  <- brewer.pal(3, "Paired")
ylab <- "Time (Seconds)"
colnames(timing) <- NULL
xpoints <- c(1, 4, 8, 12, 16)
xrange <- seq(1:17)
time <- as.vector(timing)
thread <- c(rep(1,10),rep(4,10), rep(8,10),rep(12,10),rep(16,10))
plot(x = thread, y = time, ylab = ylab, xlab = "Number of Threads", col =
    col[2], ylim=c(0,600), xaxt = "n", pch= 15)
lines(xrange, mean(timing[1])/xrange, lty = 2, lwd = 1, col = col[1])
points(xpoints, mean(timing[1])/c(1, 4, 8, 12, 16), pch = 15,cex=0.1, col = col[1])
axis(1, at = xpoints, labels = c("1", "4", "8", "12", "16"), tick = FALSE)
load("../../Result/timging_SNPNum.RData")
colnames(timing_Size_SNPs) <- NULL
barplot(c(timing_Size_SNPs[1,], timing_Size_SNPs[2,], timing_Size_SNPs[3,]),
    col=color, space=c(1, 0.1,0.1, 1, 0.1, 0.1, 1, 0.1,0.1), xlab = "SNP
    numbers", ylab=ylab,ylim=c(0,200))
axis(1, at = c(1.5+1+0.1, 2.3+4.5, 3+0.5+7.5 ), labels = format(c(200000,
     600000, 1000000),scientific=TRUE), tick = FALSE, cex = 0.3)
legend("topleft", title="Sample Size", legend = c(200, 500, 1000), col = color[1:3],
         pch = 15, bty = "n", pt.cex = 1, cex = 1.2, horiz = F, inset = c(0.1, 0.0))
dev.off()
```

Create Figure For Power

```
load("../../Result/power.RData")
setEPS()
postscript("../../Figure/Power.eps", width = 6, height = 3.5)
colors <- brewer.pal(n = 4, "Dark2")

par(mgp = c(1.9, 0.6, 0), tcl = -0.2, mar = c(4, 3, 1, 1))

plot(betaRange, Pow[, 1], ylim = c(0, 1), ylab = "Power", xlab = expression(beta),
  lty = 2, type = "l", lwd = 1.5, col=colors[1])
lines(betaRange, Pow[, 2], lty = 3, type = "l", lwd = 1.5, col=colors[2])
lines(betaRange, Pow[, 3], lty = 4, type = "l", lwd = 1.5, col=colors[3])
lines(betaRange, Pow[, 4], lty = 5, type = "l", lwd = 1.5, col=colors[4])
legend("bottomleft", title="MAF",legend =  c("0.05", "0.10", "0.20", "0.50"),
lty = c(2, 3, 4, 5), cex=1, pt.cex = 1, col = colors,bty="n" )
abline(h = 0.8, lty = 3)
graphics.off()
```

Session Information

- R version 3.4.4 (2018-03-15), `x86_64-pc-linux-gnu`
- Running under: `Ubuntu 18.04 LTS`
- Matrix products: default
- BLAS: `/usr/lib/x86_64-linux-gnu/openblas/libblas.so.3`
- LAPACK: `/usr/lib/x86_64-linux-gnu/libopenblasp-r0.2.20.so`
- Base packages: base, datasets, graphics, grDevices, methods, parallel, stats, utils
- Other packages: doParallel 1.0.11, doRNG 1.6.6, foreach 1.4.4, ggplot2 2.2.1, iterators 1.0.9, knitr 1.20, pkgmaker 0.22, RColorBrewer 1.1-2, registry 0.5, rngtools 1.2.4
- Loaded via a namespace (and not attached): codetools 0.2-15, colorspace 1.3-2, compiler 3.4.4, digest 0.6.15, evaluate 0.10.1, grid 3.4.4, gtable 0.2.0, highr 0.6, lazyeval 0.2.1, magrittr 1.5, munsell 0.4.3, pillar 1.2.2, plyr 1.8.4, Rcpp 0.12.16, rlang 0.2.0, scales 0.5.0, stringi 1.2.2, stringr 1.3.1, tibble 1.4.2, tools 3.4.4, xtable 1.8-2

```
## [1] "Start Time Fri Jun  8 11:29:16 2018"
## [1] "End Time   Fri Jun  8 11:29:18 2018"
```
