# Supplementary material for: Efficient estimation of grouped survival models
Source: BMC Bioinformatics. 2019 May 28;20:269. doi: 10.1186/s12859-019-2899-x (PMC6540566; doi:10.1186/s12859-019-2899-x)
Supplement: Supplementary file 3 — knitr-generated slides showing R code used to reproducibly conduct the analysis of the CALGB 40101 data. (PDF 145 kb) [file 12859_2019_2899_MOESM3_ESM.pdf]

CALGB40101 groupedSurv Analysis

Zhiguo Li, Jiaxing Lin, Alexander B Sibley, Tracy Truong, Katherina C Chua, Yu Jiang, Janice McCarthy, Deanna L Kroetz, Andrew Allen, and Kouros Owzar

July 19, 2018

Load R Package and Data Downloaded from dbGaP

```
library(groupedSurv)
library(GenABEL)

## Loading required package:  MASS
## Loading required package:  GenABEL.data

datadir <- "/data1/backup/avicenna/CALGB/40101/ALL/dbGaP"
wd      <- "/home/jl354/groupedsurv-manuscript"
pedfile <- file.path(datadir,'C40101_dbGaP_genotype.tped')
famfile <- file.path(datadir,'C40101_dbGaP_genotype.tfam')
phfile  <- file.path(datadir,'C40101_dbGaP_SubjectPhenotypesDD.txt')
tools::md5sum(pedfile)

## /data1/backup/avicenna/CALGB/40101/ALL/dbGaP/C40101_dbGaP_genotype.tped
##                                       "13bba0b67a7ba8bb62baaf04c398e966"

tools::md5sum(famfile)

## /data1/backup/avicenna/CALGB/40101/ALL/dbGaP/C40101_dbGaP_genotype.tfam
##                                       "0c3dfba9610a4b2eed07b4f4ca6acf2d"

tools::md5sum(phfile)

## /data1/backup/avicenna/CALGB/40101/ALL/dbGaP/C40101_dbGaP_SubjectPhenotypesDD.txt
##                                                 "9eaec39cf747a370f15b387ec553809b"
```

Convert to Genotypic Data Format

Converts genotypic data in transposed-ped format to internal genotypic data formatted file.

```
convert.snp.tped(tpedfile=pedfile, tfamfile=famfile,
                 outfile="gtdataTemp", strand="u", bcast=100000)
```

Preprocess Phynotype File

Preprocess phynotype file to the format required by function `load.gwaa.data`

```
phfileOp <- file.path(wd, "Data/C40101_dbGaP_SubjectPhenotypesDD.txt")
system(paste0("cp ", phfile," ", phfileOp))
# change " " in the tab seperated file to "_" to avoid error in load.gwaa.data.
system(paste0("sed -i 's/ /_/g' ", phfileOp))
# change Female/Male to 0 and 1 to meet the requirement of function load.gwaa.data.
system(paste0("sed -i 's/Female/0/g' ", phfileOp))
```

Create GenABEL Object

Create GenABEL object from gene data and phynotype data

```
C40101.GOBJ <- load.gwaa.data(phenofile=phfileOp,
               genofile="gtdataTemp", id='SUBJECT_ID')

## ids loaded...
## marker names loaded...
## chromosome data loaded...
## map data loaded...
## allele coding data loaded...
## strand data loaded...
## genotype data loaded...
## snp.data object created...
## assignment of gwaa.data object FORCED; X-errors were not checked!
```

Add BSA and Age phynotype to create the GenABEL object.

```
newphfile   <- file.path(wd, "Data/newphfile.tsv")
newphdata   <- read.table(newphfile, sep='\t', header=TRUE, stringsAsFactors=FALSE)
C40101.GOBJ <- add.phdata(C40101.GOBJ, newphdata)
C40101.GOBJ@gtdata@nids

## [1] 1914
```

Subset GenABEL Object for Taxol Arm

Subset the sample with BSA and Age entries and remove patient with 0 dose level.

```
C40101.Filt <- C40101.GOBJ[complete.cases(C40101.GOBJ@phdata[,c("Age","BSA")]),]
C40101.Filt <- C40101.Filt[C40101.Filt@phdata[,c("TE_Cond_CmDsBSA")]!=-2,]
C40101.Filt@gtdata@nids

## [1] 844
```

Limit analyses to autosomal SNPs

```
metadata    <- summary(C40101.Filt@gtdata)
keepChr     <- as.numeric(as.character(metadata$Chromosome)) %in% 1:22
C40101.Filt <- C40101.Filt[,keepChr]
C40101.Filt@gtdata@nsnps

## [1] 573058
```

QC-GenABEL Object from dbGaP

```
qc1 <- check.marker(C40101.Filt, maf=0.05, callrate=0.95, extr.call=0.95,
       p.level=1e-08, het.fdr=0)
```

Number of SNPs after QC

```
C40101.Filt <- C40101.Filt[,qc1$snpok]
C40101.Filt@gtdata@nsnps

## [1] 500897
```

Recover Number of Cycles from Cumulative Dose

Cycle number is determined by rounding cumulative dose to event to the nearest cycle dose (175 mg/m² per cycle)

```
breaks <- (c(0:6)+0.5)*175
C40101.Filt@phdata$Cycles <- findInterval(C40101.Filt@phdata$TE_Cond_CmDsBSA, breaks)
```

Six patients who did not experience events had dose reductions, so the number of cycles received was determined by review of patient records

```
C40101.Filt@phdata[c("IDB0140", "IDB0227", "IDB0395", "IDB0404", "IDB0788",
                     "IDB0885"), c("Cycles")] <- c(4,6,4,4,6,4)
C40101.Filt@gtdata@nids

## [1] 844
```

Preprocess Cycle Numbers

- If a patient droped out at cycle i without event, the censoring cycle of the patient should be i+1, following our cycle schema in the "Coding CALGB 40101 grouped survival data" section in SI.
- If the number of cycles are 6 and patients have no events, the event cycle numbers of the patients should be greater than 6. In our schema, the censoring cycles are treated as inf.

```
Cycles <- C40101.Filt@phdata$Cycles
Event  <- C40101.Filt@phdata$Event
Cycles[which(Event == 0)] <- Cycles[which(Event == 0)]+1
Cycles[which (Cycles==7 & Event == 0)] <- Inf
C40101.Filt@phdata$Cycles <- Cycles
table(C40101.Filt@phdata$Event,C40101.Filt@phdata$Cycles)

##
##       1   2   3   4   5   6 Inf
##   0   2  11  10   6 370   7 234
##   1  25  46  59  44  20  10   0

save(C40101.Filt, file=file.path(wd, "/Data/dbGaP_C40101_GenABEL.RData"))
```

Estimate Parameters

```
C40101.Filt@phdata$log10Age <- log10(as.numeric(C40101.Filt@phdata$Age))
Z        <- matrix(cbind(as.numeric(C40101.Filt@phdata$BSA),
                         C40101.Filt@phdata$log10Age), ncol=2)
thetaest <- thetaEst(Z, C40101.Filt@phdata$Cycles, C40101.Filt@phdata$Event)
thetaest

## $alpha
## [1] 0.9984888 0.9970534 0.9958884 0.9966530 0.9960869 0.9978534
##
## $theta
## [1] 0.6919290 0.9725007
```

Compute Efficient Score Statistics for Each SNP

```
eScore <- groupedSurv(x=NULL, Z=c("BSA","log10Age"), GenABEL.data=C40101.Filt,
    alpha=thetaest$alpha, theta=thetaest$theta, gtime=C40101.Filt@phdata$Cycles,
    delta=C40101.Filt@phdata$Event, beta=0, nCores=48)
save(eScore, file=file.path(wd, "/Result/C40101eScore_MAF.RData"))
eScoreSorted <- eScore[order(eScore$pvalue),]
eScoreSorted[1:10, ]

##                stat       pvalue       FDR      FWER
## rs12432793 23.56844 1.205483e-06 0.2394577 0.6038227
## rs17666314 23.05666 1.572971e-06 0.2394577 0.7878964
## rs9435869  22.14948 2.522246e-06 0.2394577 1.0000000
## rs11015445 21.88091 2.901061e-06 0.2394577 1.0000000
## rs4747583  21.88091 2.901061e-06 0.2394577 1.0000000
## rs1035538  21.61333 3.335255e-06 0.2394577 1.0000000
## rs11627718 20.82273 5.038154e-06 0.2394577 1.0000000
## rs9684260  20.81598 5.055929e-06 0.2394577 1.0000000
## rs7349683  20.76638 5.188590e-06 0.2394577 1.0000000
## rs6481837  20.39729 6.291879e-06 0.2394577 1.0000000
```

Save Tophit SNP IDs to File

```
topNum <- 300
write.table(rownames(eScoreSorted[1:topNum,]),
    file=file.path(wd, "/Result/C40101GroupedSurvTophit.csv"), row.names = FALSE,
    col.names = FALSE, quote=FALSE)
```

Estimating Effect Size for the Top Hits Using groupedSurv

```
library(foreach)
library(doRNG)

## Loading required package:  rngtools
## Loading required package:  pkgmaker
## Loading required package:  registry
##
## Attaching package:  'pkgmaker'
## The following object is masked from 'package:base':
##
##     isFALSE

library(doParallel)

## Loading required package:  iterators
## Loading required package:  parallel

ncores <- 48
registerDoParallel(cores=ncores)
TopHitSNPID <- rownames(eScoreSorted[1:topNum,])
betaest <- foreach(i=1:topNum, .combine=rbind)%dopar%{
  x <- matrix(as.numeric(C40101.Filt@gtdata[,TopHitSNPID[i]]), ncol=1)
  res <- betaEst(x=x, Z=Z, alpha=thetaest$alpha, theta=thetaest$theta,
      gtime=C40101.Filt@phdata$Cycles,
      delta=C40101.Filt@phdata$Event)
  return(res)
}
colnames(betaest) <- c("beta")
rownames(betaest) <- TopHitSNPID
```

Anotate the Top Hits

- The list of top SNPs is annotated with chromosome and position info from `SNPlocs.Hsapiens.dbSNP150.GRCh38`.

Annotate top hits.

```
source("./promotor.R")
```

Compute MAF for the dataset

```
MAF  <- summary(C40101.Filt)$Q.2
MAF <- as.data.frame(MAF, ncol=1)
rownames(MAF) <- rownames(eScore)
```

Anotate the Top Hits (Continues...)

SNP ids did not get annotated

```
TopHitSNPID[!TopHitSNPID %in% rownames(rawAnno)]

## [1] "rs12191315" "rs2169100"  "rs1928078"  "rs2827050"  "rs2479996"
## [6] "rs988831"   "rs4818638"
```

Export annotation and write to file.

```
rawAnno <- rawAnno[TopHitSNPID,]
LRGene <- paste0(rawAnno$LEFTSYMBO, "/",rawAnno$RIGHTSYMBOL)
Gene <- rawAnno$GENESYMBOL
Gene[which(is.na(Gene))] <- LRGene[which(is.na(Gene))]
TopHitAno <- data.frame(rsID=TopHitSNPID, Chr=rawAnno$seqnames,
    Pos=rawAnno$start, Gene=Gene, LOCATION=rawAnno$LOCATION,
    MAF=MAF[TopHitSNPID,1])
rownames(TopHitAno) <- TopHitAno$rsID
TopHitAno$Pval <- eScoreSorted[TopHitSNPID, 2]
TopHitAno <- cbind(TopHitAno, beta=betaest)
write.csv(TopHitAno, file=file.path(wd, "/Result/C40101GroupedSurvTopHitAnno.csv"),
    row.names = FALSE, quote=FALSE)
```

Session Information

- R version 3.5.1 (2018-07-02), `x86_64-pc-linux-gnu`
- Running under: `Ubuntu 18.04 LTS`
- Matrix products: default
- BLAS: `/usr/lib/x86_64-linux-gnu/openblas/libblas.so.3`
- LAPACK: `/usr/lib/x86_64-linux-gnu/libopenblasp-r0.2.20.so`
- Base packages: base, datasets, graphics, grDevices, methods, parallel, stats, stats4, utils
- Other packages: AnnotationDbi 1.42.1, Biobase 2.40.0, BiocGenerics 0.26.0, BiocParallel 1.14.2, Biostrings 2.48.0, BSgenome 1.48.0, DelayedArray 0.6.1, doParallel 1.0.11, doRNG 1.7.1, foreach 1.4.4, GenABEL 1.8-0, GenABEL.data 1.0.0, GenomeInfoDb 1.16.0, GenomicFeatures 1.32.0, GenomicRanges 1.32.4, groupedSurv 1.0.3, IRanges 2.14.10, iterators 1.0.10, knitr 1.20, MASS 7.3-50, matrixStats 0.53.1, org.Hs.eg.db 3.6.0, pkgmaker 0.27, registry 0.5, rngtools 1.3.1, Rsamtools 1.32.2, rtracklayer 1.40.3, S4Vectors 0.18.3, SNPlocs.Hsapiens.dbSNP150.GRCh38 0.99.20, SummarizedExperiment 1.10.1, TxDb.Hsapiens.UCSC.hg38.knownGene 3.4.0, VariantAnnotation 1.26.1, XVector 0.20.0
- Loaded via a namespace (and not attached): assertthat 0.2.0, bibtex 0.4.2, bindr 0.1.1, bindrcpp 0.2.2, biomaRt 2.36.1, bit 1.1-14, bit64 0.9-7, bitops 1.0-6, blob 1.1.1, codetools 0.2-15, colorspace 1.3-2, compiler 3.5.1, crayon 1.3.4, DBI 1.0.0, digest 0.6.15, dplyr 0.7.6, evaluate 0.10.1, GenomeInfoDbData 1.1.0, GenomicAlignments 1.16.0, ggplot2 3.0.0, glue 1.2.0, grid 3.5.1, gtable 0.2.0, highr 0.7, hms 0.4.2, httr 1.3.1, lattice 0.20-35, lazyeval 0.2.1, magrittr 1.5, Matrix 1.2-14, memoise 1.1.0, munsell 0.5.0, pillar 1.3.0, pkgconfig 2.0.1, plyr 1.8.4, prettyunits 1.0.2, progress 1.2.0, purrr 0.2.5, qvalue 2.12.0, R6 2.2.2, Rcpp 0.12.17, RCurl 1.95-4.10, reshape2 1.4.3, rlang 0.2.1, RSQLite 2.1.1, scales 0.5.0, splines 3.5.1, stringi 1.2.3, stringr 1.3.1, tibble 1.4.2, tidyselect 0.2.4, tools 3.5.1, withr 2.1.2, XML 3.98-1.11, xtable 1.8-2, zlibbioc 1.26.0

```
## [1] "Start Time Thu Jul 19 11:38:46 2018"
## [1] "End Time   Thu Jul 19 11:47:53 2018"
```
